# Supplementary material for: Loss of ALK4 promotes cancer progression through regulating TGF-β receptor N-glycosylation
Source: Nat Commun. 2025 Dec 17;17:854. doi: 10.1038/s41467-025-67563-1 (PMC12828005; doi:10.1038/s41467-025-67563-1)
Supplement: Supplementary file 1 — Supplementary Information [file 41467_2025_67563_MOESM1_ESM.pdf]

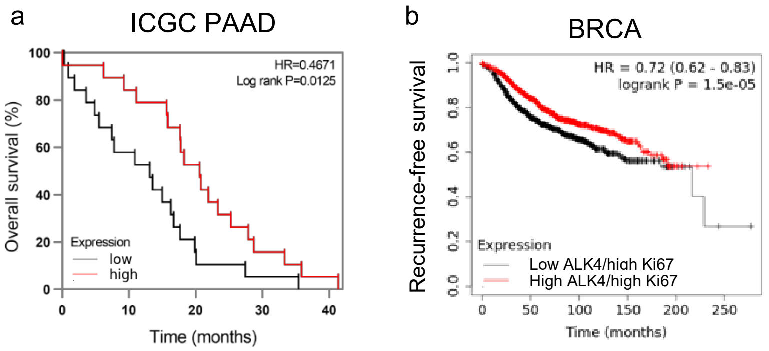

Supplementary Figure 1. Low ACVR1B expression correlates with poorer prognosis in pancreatic and breast cancer cohorts. a Kaplan–Meier plot of overall survival for pancreatic cancer patients stratified by ACVR1B expression (20% low vs. 20% high cutoff) in the ICGC PAAD-AU dataset (n=95). b Kaplan–Meier plots of recurrence-free survival (n=4929) for breast cancer patients with high Ki67 proliferation indices, stratified by ACVR1B expression at the median.

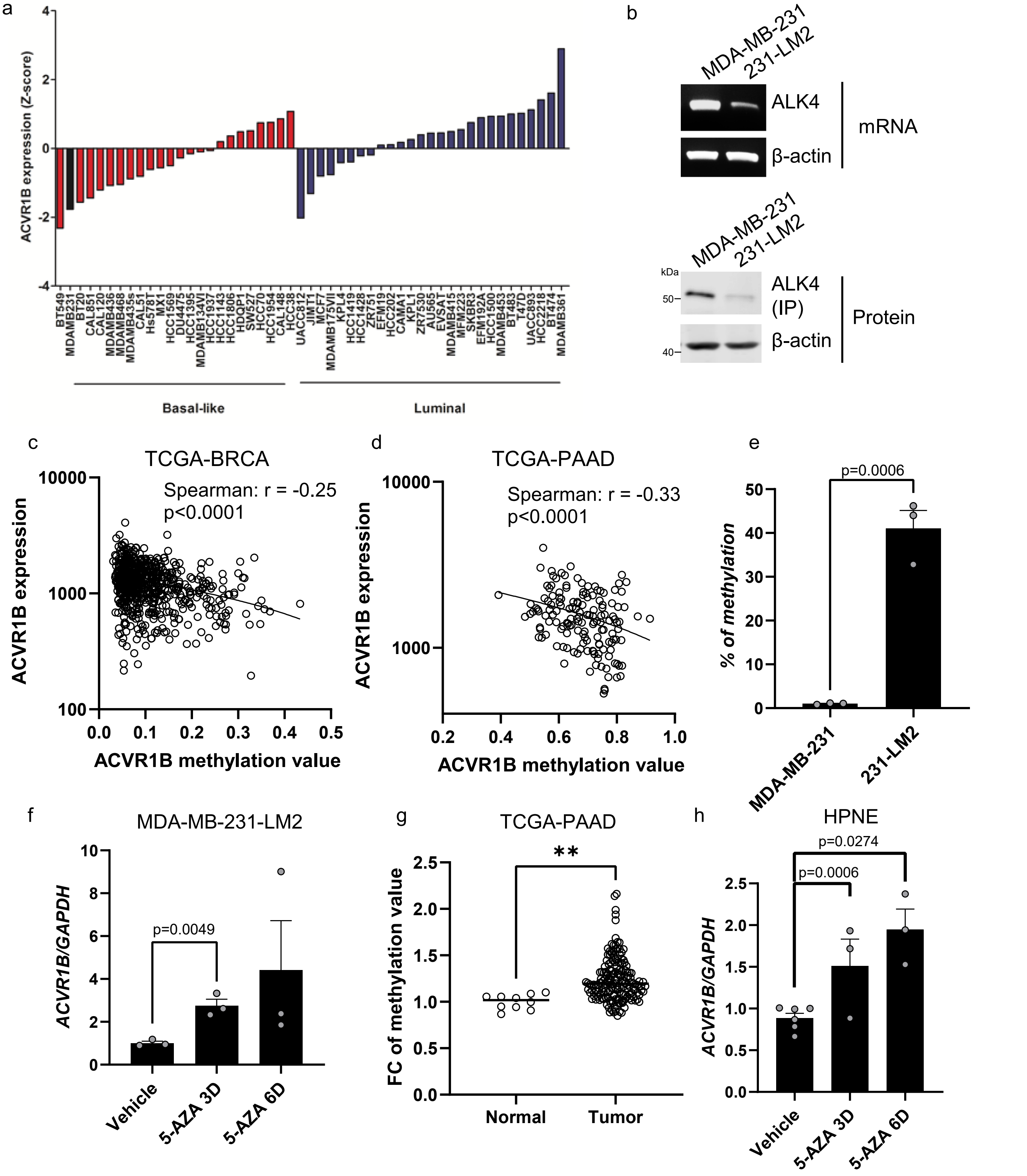

Supplementary Figure 2. Epigenetic regulation of ACVR1B in pancreatic and breast cancer. a ACVR1B expression across 51 human breast cancer cell lines. **b** ALK4 mRNA and protein levels in MBA-MB-231 and LM2 cells. **c–d** Correlation between ACVR1B expression and ACVR1B methylation levels in breast cancer patients (n=656) (**c**) and pancreatic cancer patients (n=149) (**d**), based on cBioportal TCGA data. Correlation analysis was performed using nonparametric two-tailed Spearman correlation. **e** Percentage of ACVR1B methylation in MDA-MB-231 and MDA-MB-231-LM2 cells (n=3 per group). **f** qRT-PCR assessment of ACVR1B expression in MDA-MB-231-LM2 cells (n=3 per group) treated with 5 μM 5-Azacitidine for 3 days (3D) or 5 days (5D). **g** Fold change in ACVR1B methylation between TCGA normal tissues (n=10) and pancreatic cancer samples (n=185). **h** ACVR1B expression in HPNE NTC (n=6) and 5-AZA treated cells (n=3). For in vitro analysis, each experiment was done with 3 independent biological replicates. Statistical analysis was performed using two-tailed Student's t-tests for comparisons or nonparametric two-tailed Spearman correlation for associations. Data are presented as mean values +/- SEM.

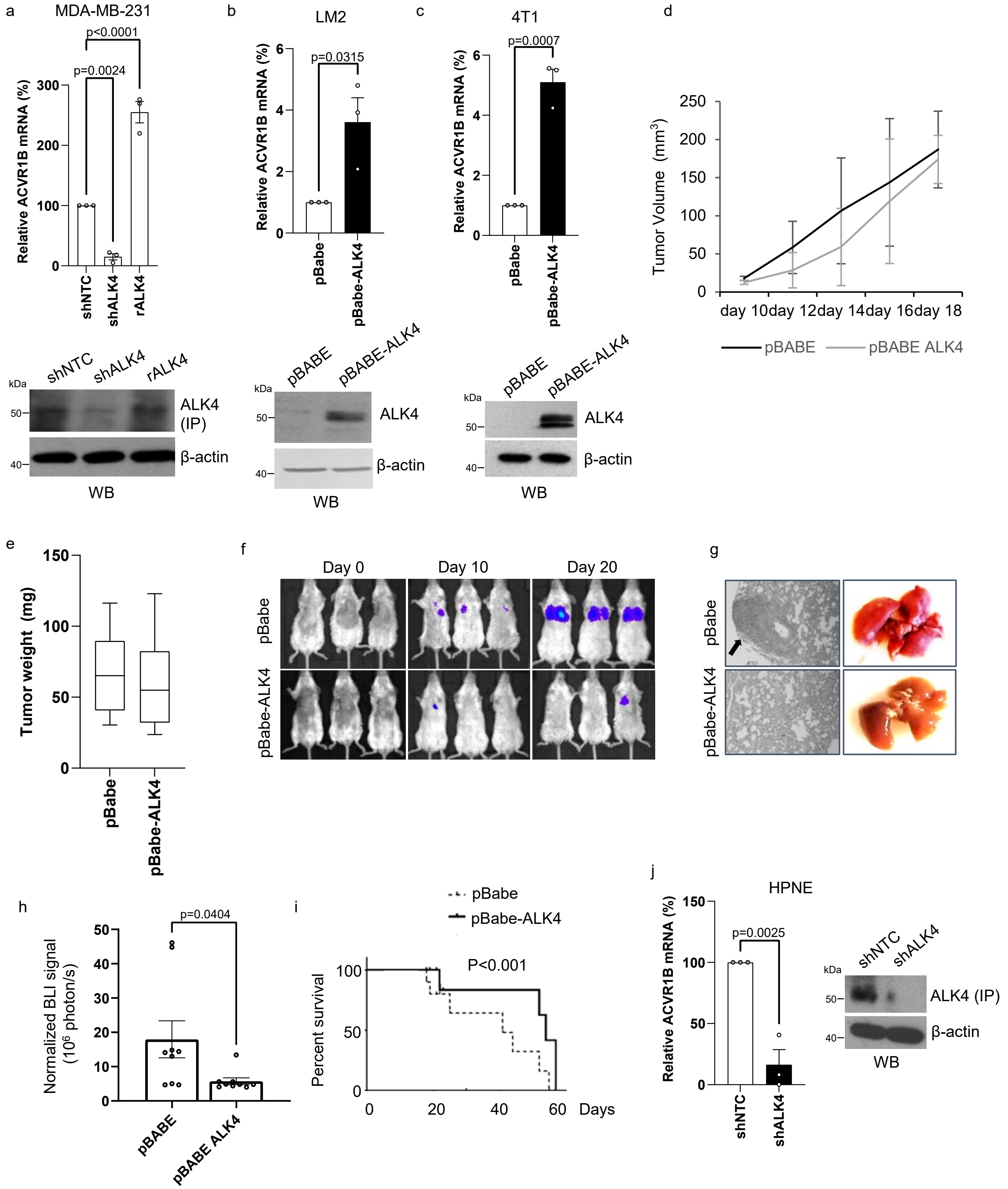
a
MDA-MB-231
p<0.0001
p=0.0024
Relative ACVR1B mRNA (%)
300
200
100
0
shNTC
shALK4
rALK4
kDa
50
40
ALK4
(IP)
β-actin
WB
b
LM2
p=0.0315
Relative ACVR1B mRNA (%)
6
4
2
0
pBabe
pBabe-ALK4
pBABE
pBABE-ALK4
ALK4
β-actin
WB
c
4T1
p=0.0007
Relative ACVR1B mRNA (%)
pBabe
pBabe-ALK4
pBABE
pBABE-ALK4
ALK4
β-actin
WB
d
Tumor Volume (mm^3)
250
200
150
100
50
0
day 10
day 12
day 14
day 16
day 18
pBABE
pBABE ALK4
e
Tumor weight (mg)
150
100
50
0
pBabe
pBabe-ALK4
f
Day 0
Day 10
Day 20
pBabe
pBabe-ALK4
g
pBabe
pBabe-ALK4
h
p=0.0404
Normalized BLI signal
(10^6 photon/s)
50
40
30
20
10
0
pBABE
pBABE ALK4
i
pBabe
pBabe-ALK4
P<0.001
Percent survival
100
50
0
0
20
40
60
Days
j
HPNE
p=0.0025
Relative ACVR1B mRNA (%)
150
100
50
0
shNTC
shALK4
kDa
50
40
ALK4 (IP)
β-actin
WB

Supplementary Figure 3. Restoring ALK4 expression decreases breast cancer progression. a qRT-PCR analysis of ACVR1B mRNA levels in MDA-MB-231 shNTC, shALK4, or shALK4 with shRNA-resistant ALK4 cells (n=3 per group). ALK4 protein levels were assessed using immunoprecipitation (lower panel). **b** qRT-PCR analysis of ALK4 mRNA levels in LM2 pBabe or pBabe-ALK4 cells (n=3 per group). ALK4 protein levels were assessed by Western blotting (lower panel). **c** qRT-PCR analysis of ALK4 mRNA levels in 4T1 pBabe or pBabe-ALK4 cells (n=3 per group). ALK4 protein levels were assessed by Western blotting (lower panel). **d** Tumor volume of 4T1 cells expressing pBabe or pBabe-ALK4 orthotopically injected into the mammary fat pad was calculated as Volume=1/2 L*W2 where tumor length and width were measured with a caliper. **e** Average primary tumor weight at excision on day 18. **f** Lung metastases were monitored by bioluminescence imaging at the indicated days after primary tumor excision, with representative images provided. **g** Lung sections were stained with H&E, with representative whole-lung and section images shown. Metastases are indicated by black arrow. **h** Quantification of bioluminescent signals for lung metastases on day 20, normalized to baseline. **i** Kaplan–Meier survival curves for mice injected with 4T1 cells expressing pBabe or pBabe-ALK4 following removal of primary mammary tumors. **j** qRT-PCR analysis of ACVR1B mRNA levels in HPNE cells shNTC or shALK4 cell (n=3 per group). ALK4 protein levels were assessed by immunoprecipitation (lower panel). For in vitro analysis, each experiment was done with at least 3 independent biological replicates. Data were analyzed using two-tailed Student’s t-tests for comparisons between two groups. Data are presented as mean values +/- SEM.

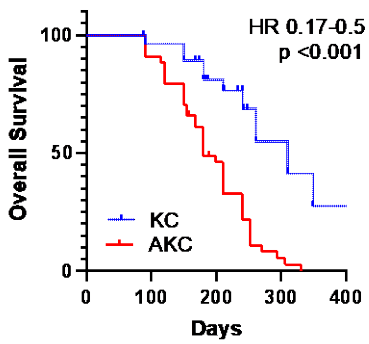

b

	PDAC Incidence (%)	Ascites Incidence (%)
KC	3/10 (30%)	1 of 34 (3%)
AKC	10/10 (100%)	7 of 33 (21%)
P value	0.003	0.03

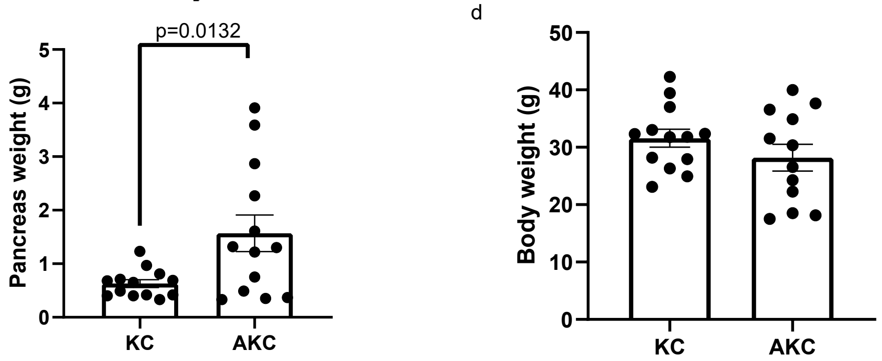

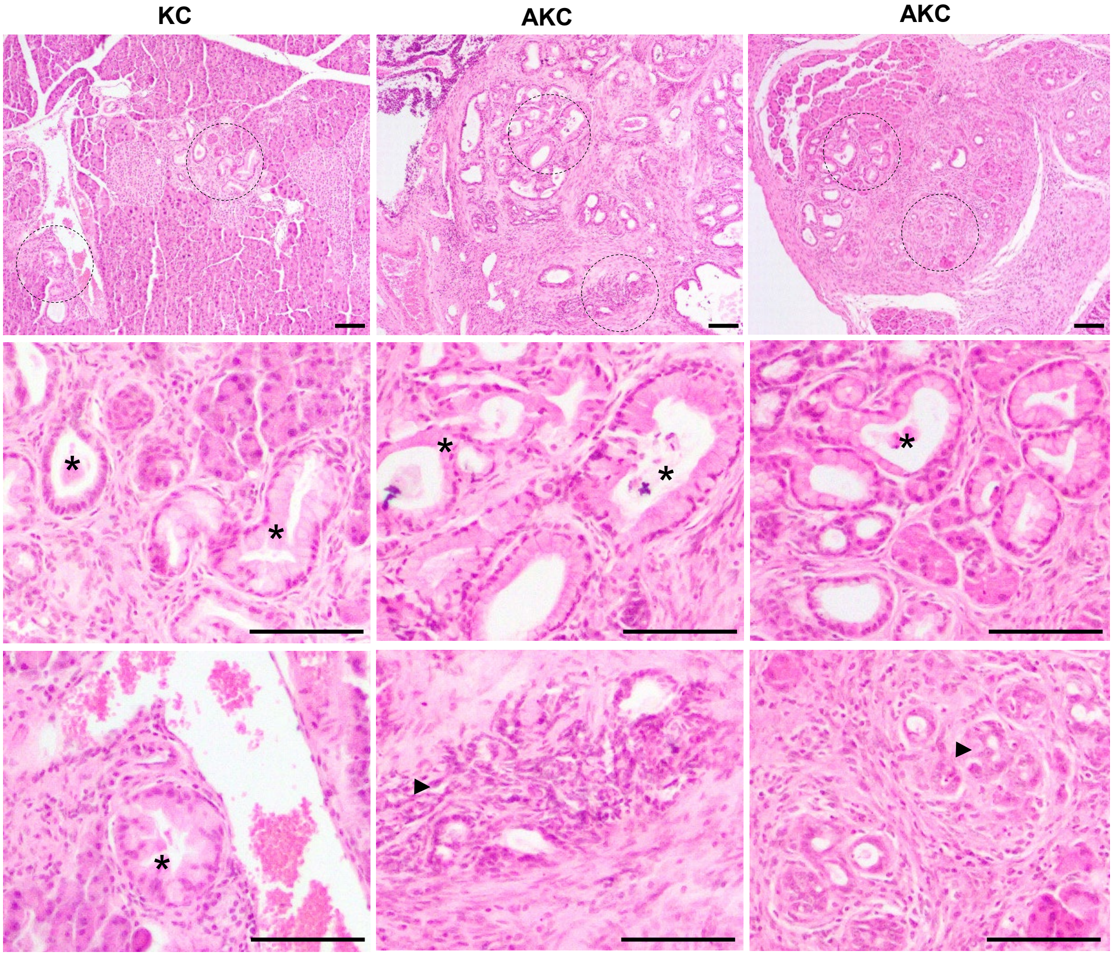

Supplementary Figure 4. Loss of Acvr1b in KrasG12D-mutated mouse pancreata decreases overall survival and promotes pancreatic intraepithelial neoplasias (PanINs) and pancreatic ductal adenocarcinoma (PDAC) progression. a Kaplan–Meier overall survival curves of KC (KRAS^{G12D}; Pdx1-cre) and AKC (ALK4$^{fl/fl}$; KRAS^{G12D}; Pdx1-cre) mice (KC: n=29; AKC: n=46). **b** Incidence and percentage of mice that developed PDAC in KC (n=10) and AKC (n=10) mice at 8–9 months of age; incidence and percentage of mice observed with ascites at humane end point or by the time of euthanasia in KC (n=34) and AKC (n=33) mice. Results were analyzed using the Mann–Whitney U test. **c** Pancreatic weights in KC and AKC mice at 8-9 months of age (n=13 per group). **d** Body weights of KC and AKC mice at 8-9 months of age (AKC: n=12; KC: n=13). **e** Representative images of pancreatic lesions in KC and AKC mice. * indicates PanIN lesions and arrowheads indicate intermediate/poorly differentiated ducts. Pancreatic sections were evaluated by a board-certified veterinary pathologist in a blinded manner. Scale bar=100 μm. Data were analyzed using two-tailed Student's t-tests for comparisons between two groups. Data are presented as mean values +/- SEM.

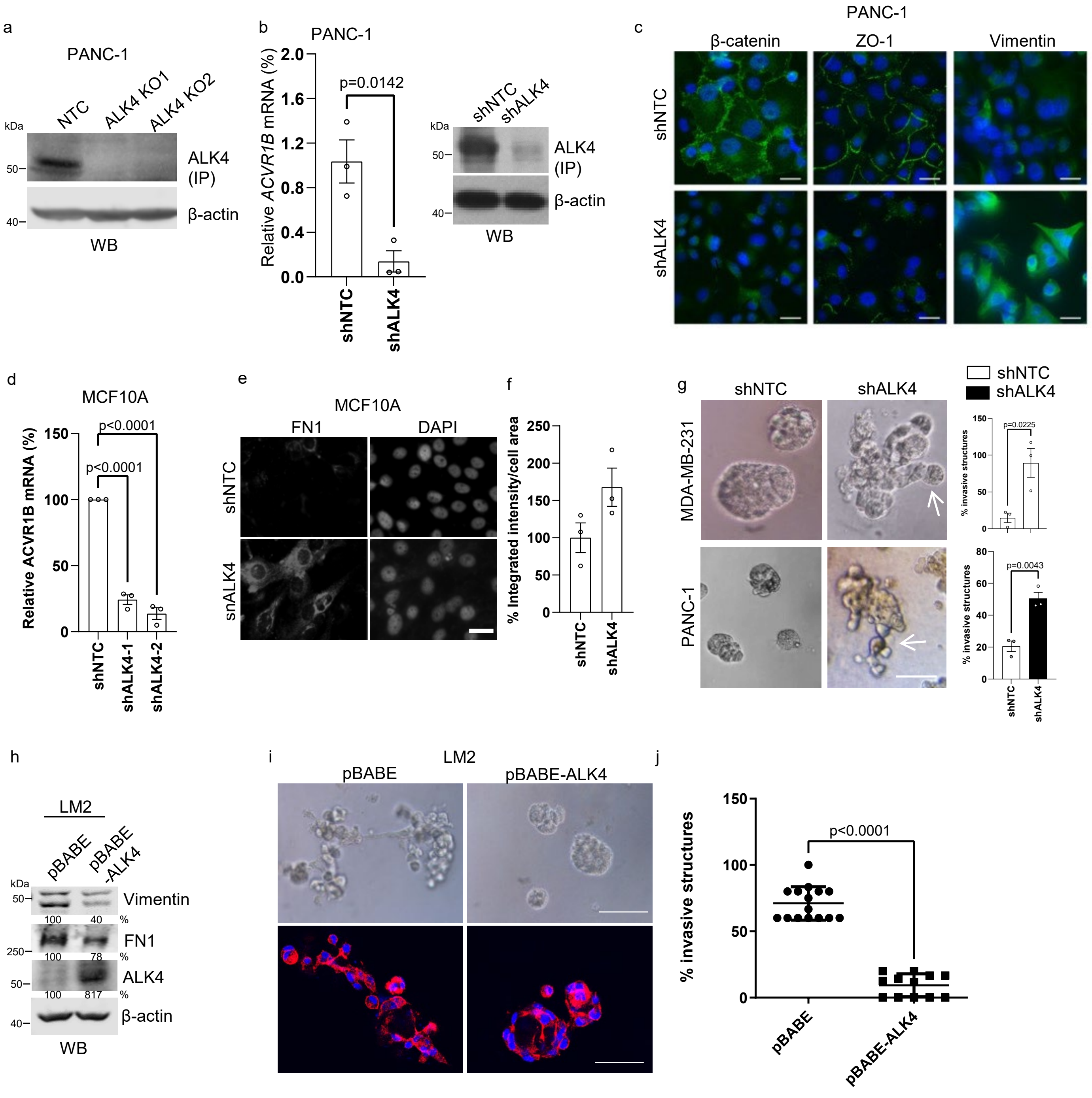

Supplementary Figure 5. Loss of ALK4 expression promotes cancer progression. a ALK4 protein levels in PANC-1 cells with CRISPR NTC and ALK4 CRISPR KO were determined via immunoprecipitation and Western blotting. **b** ACVR1B mRNA levels in PANC-1 cells expressing shNTC or shALK4 (n=3 per group), and ALK4 protein levels were analyzed by immunoprecipitation and western blotting (right). **c** Expression and localization of ZO-1, β-catenin, and vimentin in PANC-1 cells stably expressing shNTC or shALK4 were assessed by immunofluorescent staining. Scale bar=50 μm. **d** ACVR1B mRNA levels in MCF10A shNTC or two shRNAs targeting ALK4 cells (n=3 per group) were quantified by qRT-PCR. **e** MCF10A cells expressing shNTC or shALK4 were fixed, stained for fibronectin (FN1), counterstained with DAPI, and imaged under a fluorescence microscope. Scale bar=50 μm. **f** FN1 intensity of panel e was quantitated using ImageJ. **g** MDA-MB-231 and PANC-1 cells expressing shNTC or shALK4 were subjected to a Matrigel 3D culture assay. Extended structures were considered invasive structures (white arrows). Scale bar=100 μm. **h** Expression of mesenchymal markers (vimentin and FN1) in LM2 cells stably expressing pBABE or pBABE-ALK4 was analyzed by Western blotting. **i** LM2 cells expressing pBABE or pBABE-ALK4 were subjected to a Matrigel 3D culture assay. Representative images of invasive structures were captured using light microscopy or phalloidin staining for F-actin after 8 days. Scale bar=200 μm. **j** The invasive structures of panel i were quantified. For in vitro analysis, each experiment was done with at least 3 independent biological replicates. Data were analyzed using two-tailed Student's t-tests for comparison between two groups. Data are presented as mean values +/- SEM.

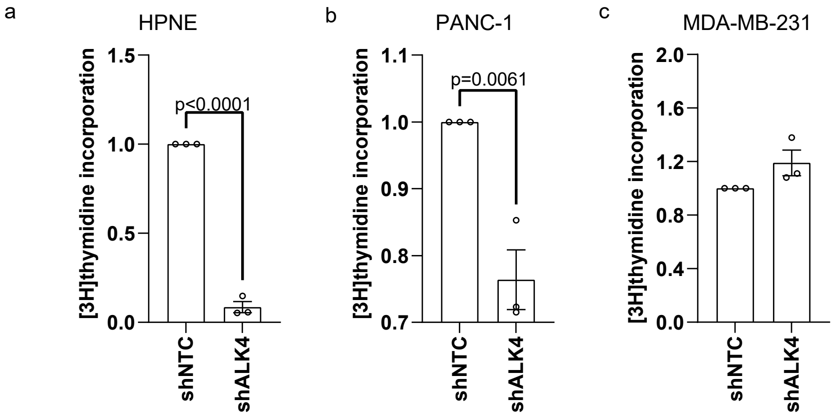

Supplementary Figure 6. Loss of ALK4 does not increase cell proliferation. a–c HPNE cells expressing shNTC or shALK4 (**a**), PANC-1 cells expressing shNTC or shALK4 (**b**), MDA-MB-231 cells expressing shNTC or shALK4 (**c**) were subjected to 3H thymidine incorporation assays. Normalized incorporation levels are plotted. Each experiment was done with 3 independent biological replicates. Data were analyzed using two-tail Student's t-tests for comparison between two groups. Data are presented as mean values +/- SEM.

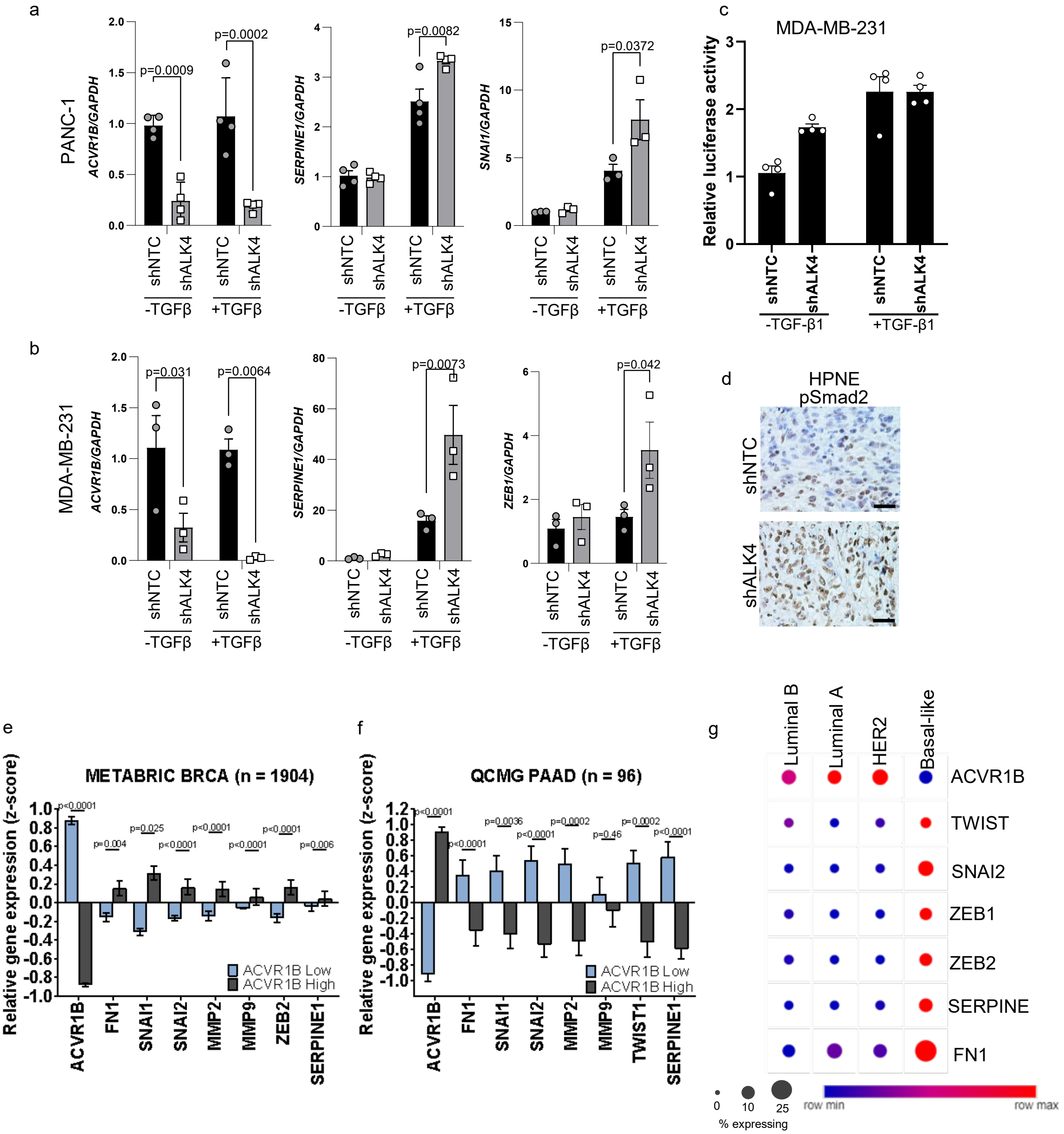

Supplementary Figure 7. Loss of ALK4 expression correlates with increased expression of TGF-β target genes in breast and pancreatic cancer. a–b PANC-1 (a) or MDA-MB-231 (b) cells stably expressing shNTC or shALK4 (n=3 per group) were serum-starved for 3 h and treated with 100 pM TGF-β1 for 6 h. RNA was extracted, and TGF-β target gene expression was analyzed by qRT-PCR. **c** MDA-MB-231 cells expressing shNTC or shALK4 (n=4 per group), along with the Smad2/3-binding pE2.1 luciferase reporter and Renilla luciferase, were serum-starved for 3 h, treated with vehicle or 100 pM TGF-β1 for 30 min, and subjected to a dual luciferase reporter assay. **d** HPNE cells expressing shNTC or shALK4 were orthotopically injected into the tail of mouse pancreata (same cohort as in Figure 2g-k). Primary tumors were sectioned and stained with a phospho-Smad2 antibody. Scale bar=50 μm. **e** TGF-β target gene expression negatively correlated with ACVR1B expression in the cBioPortal METABRIC breast cancer dataset (n=1904). **f** TGF-β target gene expression negatively correlated with ACVR1B expression in the cBioPortal QCMG pancreatic cancer dataset (n=96). **g** Dot plot showing the proportion of cells in respective K-means clusters expressing each gene (dot size) and average expression levels (color scale). Data were from the Single Cell Portal (Broad Institute). For in vitro analysis, each experiment was done with 3 independent biological replicates. For experiments with two independent variables, data were analyzed using ordinary two-way ANOVA followed by Tukey's multiple comparisons test. For comparison between two groups, data were analyzed using two-tail Student’s t tests. Data are presented as mean values +/- SEM.

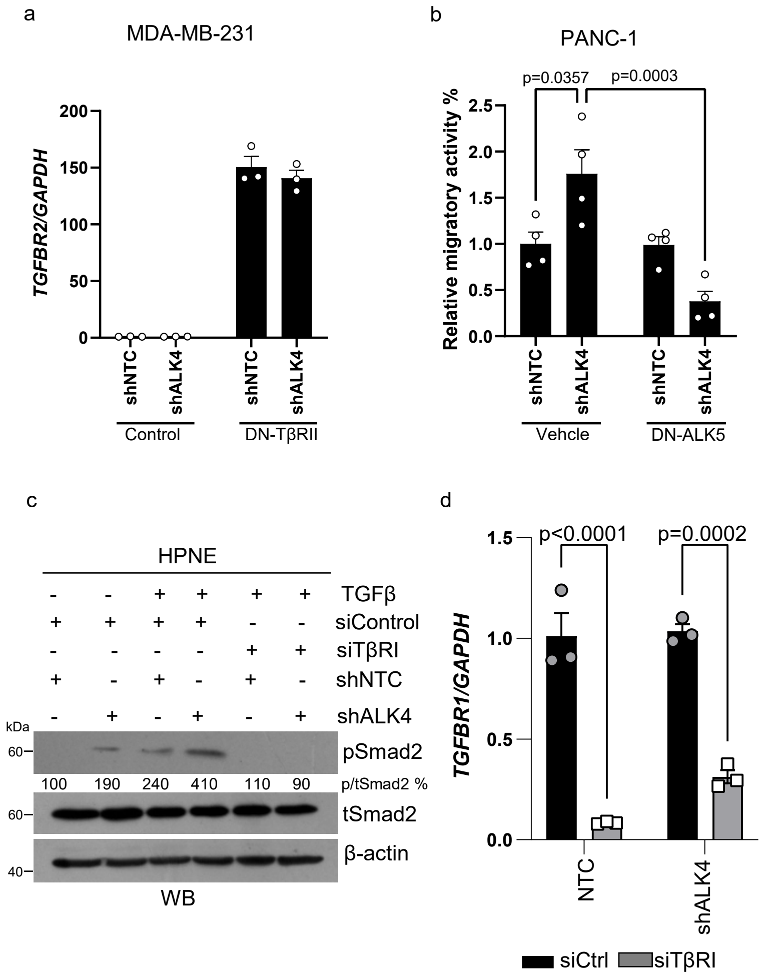

Supplementary Figure 8. TGF-β signaling mediates the downstream effects of ALK4 loss. a TGFBR2 (TβRII) mRNA levels in MDA-MB-231 shNTC or shALK4 cells (n=3 per group) and transfected with a control plasmid or dominant-negative TβRII (DN-TβRII) plasmid were analyzed by qRT-PCR. **b** transwell migration assay of PANC-1 cells stably expressing shNTC or shALK4 (n=4 per group). Cells were transfected with DN-ALK5 for 48 h and allowed to migrate toward 10% FBS for 24 h. Migration values are presented as mean ± SD. **c** HPNE cells stably expressing shNTC or shALK4 were transfected with non-targeting control or TβRI siRNA for 48 h. Cells were serum-starved for 3 h, treated with 100 pM TGFβ for 30 min, and subjected to Western blot analysis pSmad2, total-Smad2, and β-actin. **d** TGFBR1 (TβRI) mRNA levels were analyzed by qRT-PCR in HPNE shNTC and shALK4 cells (n=3 per group) transfected with siCtrl or siTβRI from (c). Each experiment was done with 3 independent biological replicates. For experiments with two independent variables, data were analyzed using ordinary two-way ANOVA followed by Tukey's multiple comparisons test. Data are presented as mean values +/- SEM.

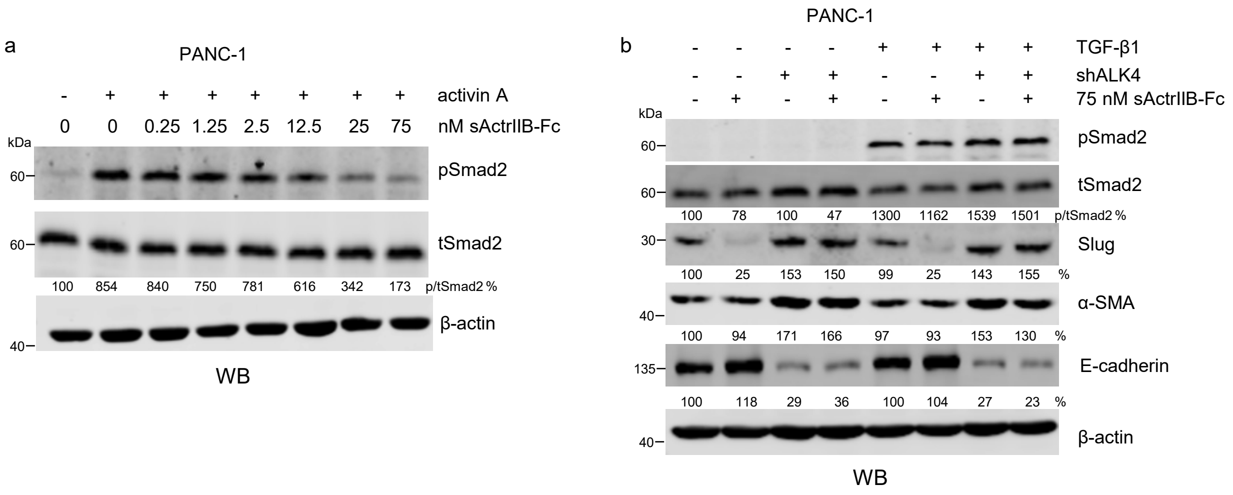

Supplementary Figure 9. Loss of ALK4 increases TGF-β signaling and EMT independent of Activin A signaling. a PANC-1 cells were treated with sActrIIB-Fc for 72 h, followed by 30 min of activin A treatment. Protein lysates were analyzed by Western blotting for pSmad2, total Smad2, and β-actin. **b** PANC-1 cells expressing control shRNA or ALK4 shRNA were treated with sActrIIB-Fc for 72 h, followed by 30 min of TGF-β1 treatment. Protein lysates were analyzed by Western blotting for indicated proteins. Each experiment was done with 3 independent biological replicates.

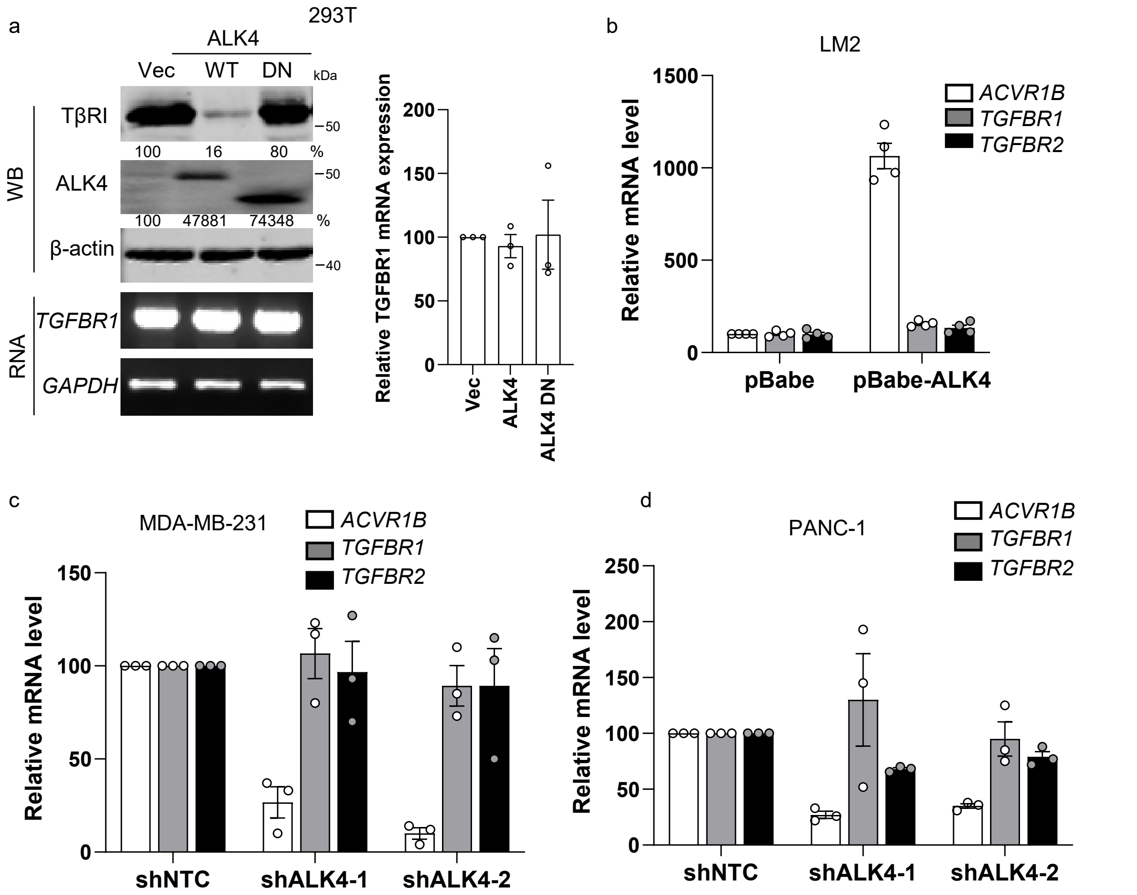

Supplementary Figure 10. ALK4 negatively regulates TGF-β signaling without affecting TGF-β receptors' mRNA level. a 293T cells transfected with pcDNA3.1, WT ALK4, or DN ALK4 plasmids for 48 h (n=3 per group). Protein lysates and total RNA were extracted and assessed using western blotting and qRT-PCR, respectively. **b** qRT-PCR analysis of TGFBR1, TGFBR2, and ACVR1B in LM2 cells expressing pBABE or pBABE-ALK4 (n=4 per group). c–d qRT-PCR analysis of TGFBR1, TGFBR2, and ALK4 in MDA-MB-231 (**c**) and PANC-1 (**d**) cells stably expressing shNTC, shALK4-1, or shALK4-2 (n=3 per group). Each experiment was done with 3 independent biological replicates. Data are presented as mean values +/- SEM.

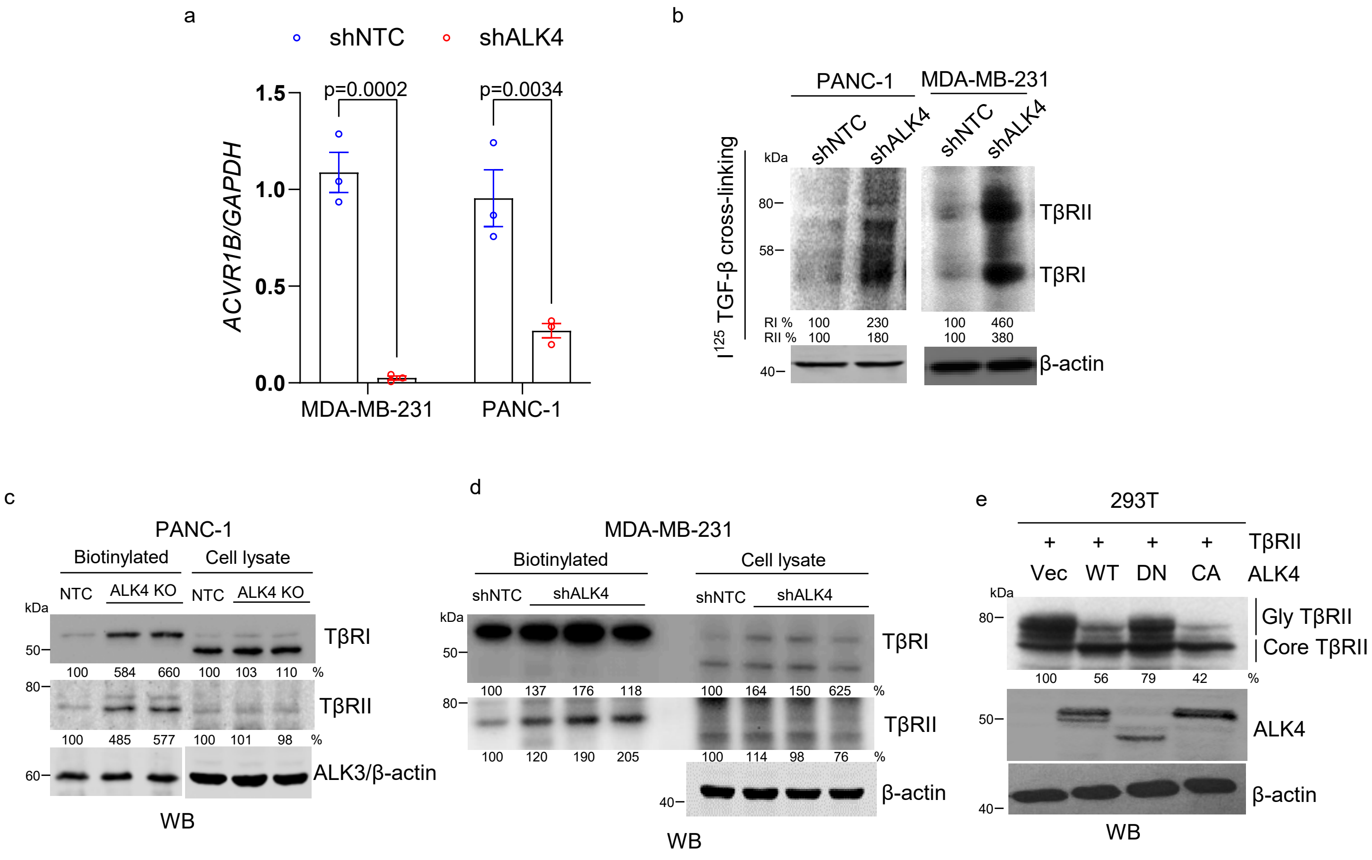

Supplementary Figure 11. ALK4 loss increases cell surface expression of TGF-β receptors. a qRT-PCR analysis of ACVR1B mRNA expression in MDA-MB-231 or PANC-1 shNTC and shALK4 cells (n=3 per group). **b** PANC-1 and MDA-MB-231 cells expressing shNTC or shALK4 were serum-starved for 3 h and treated with 100 pM TGF-β1 for 30 min. Cell surface levels of TGF-β receptors were assessed using an I125-TGF-β binding and crosslinking assay. **c-d** Total protein from whole-cell lysates or biotinylated cell surface proteins from PANC-1 NTC and ALK4 KO cells (**c**) and MDA-MB-231 shNTC and shALK4 cells (**d**) were analyzed for TβRI and TβRII by Western blotting. ALK3/β-actin (**c**) or β-actin (**d**) were used as loading controls. **e** 293T cells were transfected with expression plasmids for TβRII, along with control, WT, CA, or DN ALK4. TβRII, ALK4, and β-actin were assessed by Western blotting. Each experiment was done with 3 independent biological replicates. Data are presented as mean values +/- SEM.

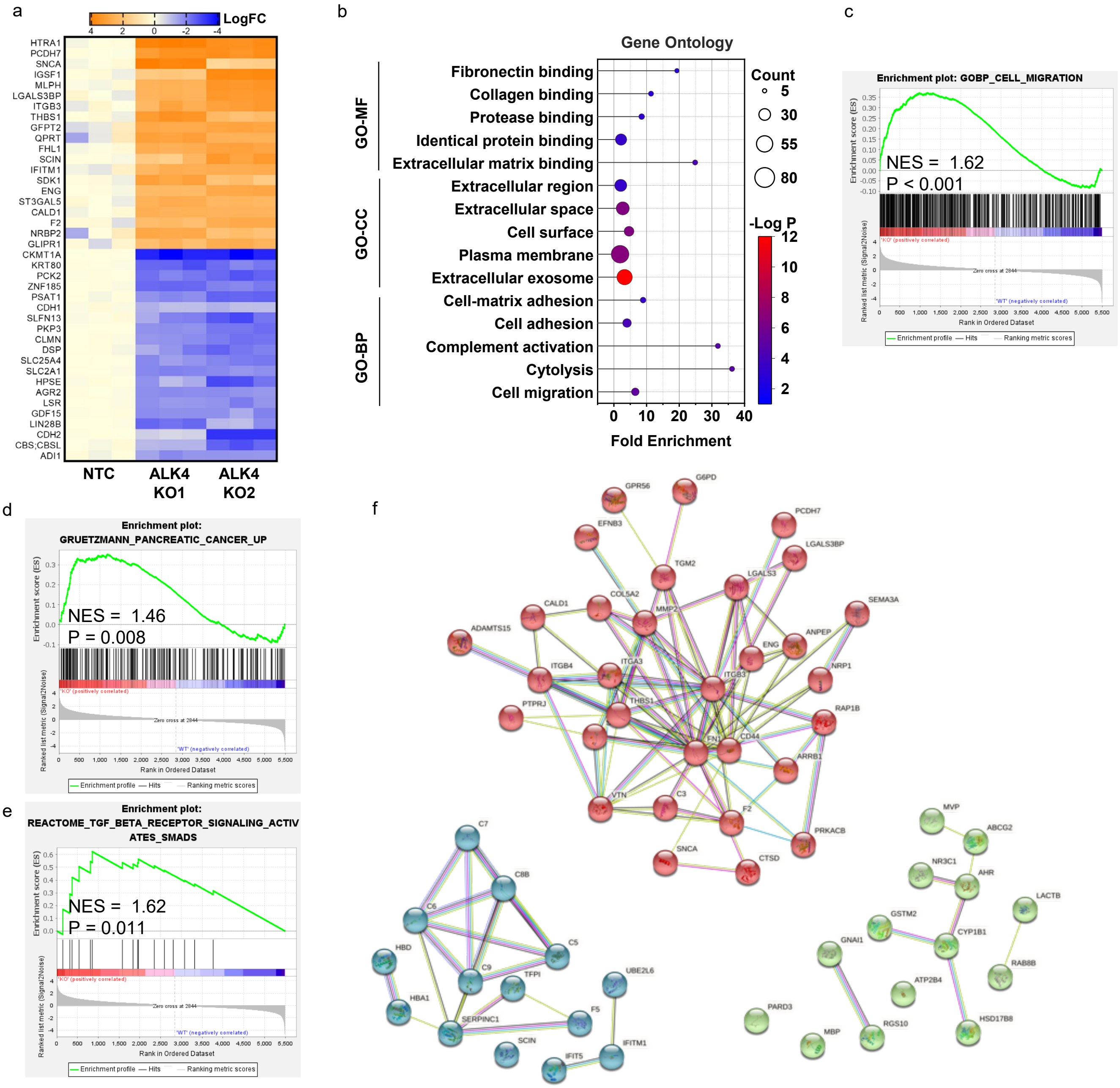

Supplementary Figure 12. Detailed proteomic analysis of ALK4 loss reveals altered gene expression, functional enrichment, and protein interactions. a Heatmap showing gene expression patterns of the top 20 most upregulated and top 20 most downregulated differentially expressed proteins (DEPs, KO vs Ctrl). Expression values are represented as colors, ranging from orange (high expression) to blue (low expression). **b** Gene ontology enrichment analysis of the top 125 upregulated genes in ALK4 KO compared to control samples, performed using DAVID Bioinformatics. The most significant gene sets are shown. **c–e** Gene set enrichment analysis (GSEA) enrichment plots for gene signatures significantly and positively enriched in the ALK4 KO group. Normalized enrichment scores (NES) and p-values are shown for each plot. **f** Cluster analysis and protein-protein interaction network of the top 125 upregulated genes in ALK4 KO samples using STRING. Interactions with a score greater >0.5 are shown. Three distinct clusters are identified and colored red, blue, and green. Solid lines indicate interactions within the same cluster. Interaction types are indicated by line color (cyan-curated databases; pink-experimentally determined; Blue-gene co-occurrence; khaki-text mining; black-co-expression; light blue-protein homology).

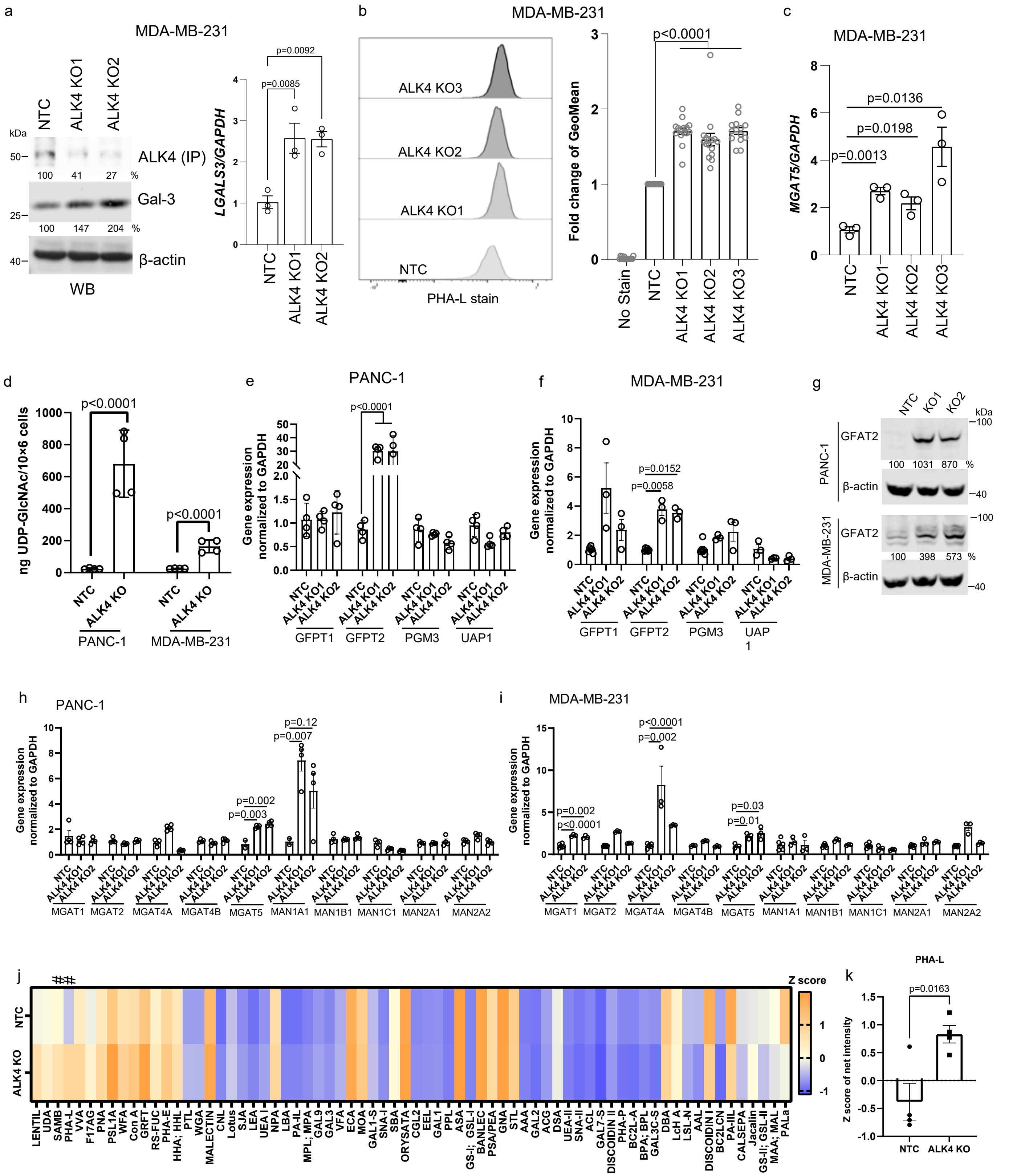
a
MDA-MB-231
NTC
ALK4 KO1
ALK4 KO2
kDa
ALK4 (IP)
100 41 27 %
Gal-3
100 147 204 %
β-actin
WB
LGALS3/GAPDH
p=0.0092
p=0.0085
b
MDA-MB-231
ALK4 KO3
ALK4 KO2
ALK4 KO1
NTC
PHA-L stain
Fold change of GeoMean
p<0.0001
No Stain
c
MDA-MB-231
MGAT5/GAPDH
p=0.0136
p=0.0198
p=0.0013
ALK4 KO3
d
ng UDP-GlcNAc/10×6 cells
p<0.0001
ALK4 KO
PANC-1
MDA-MB-231
e
PANC-1
Gene expression normalized to GAPDH
p<0.0001
GFPT1
GFPT2
PGM3
UAP1
f
MDA-MB-231
p=0.0152
p=0.0058
UAP 1
g
KO1
KO2
GFAT2
100 1031 870 %
100 398 573 %
h
PANC-1
p=0.12
p=0.007
p=0.002
p=0.003
MGAT1
MGAT2
MGAT4A
MGAT4B
MGAT5
MAN1A1
MAN1B1
MAN1C1
MAN2A1
MAN2A2
i
p<0.0001
p=0.002
p=0.03
p=0.01
j
Z score
LENTIL
UDA
SAMB
PHA-L
VVA
F17AG
PNA
PSL1A
WFA
Con A
GRFT
RS-FUC
PHA-E
HHA; HHL
PTL
WGA
MALECTIN
CNL
Lotus
SJA
LEA
UEA I
NPA
LBA
PA-IL
MPL; MPA
GAL9
GAL3
VFA
ECA
MOA
GAL1-S
SNA-I
SBA
ORYSATA
CGL2
EEL
GAL1
PPL
ASA
GS-I; GSL-I
BANLEC
PSA/PEA
GNA
STL
AAA
GAL2
ACG
DSA
UEA-II
SNA-II
ACL
GAL7-S
DISCOIDIN II
PHA-P
BC2L-A
BPA; BPL
GAL3C-S
DBA
LcH A
LSL-N
AAL
DISCOIDIN I
BC2LCN
PA-IIL
CALSEPA
Jacalin
GS-II; GSL-II
MAA; MAL
PALa
k
PHA-L
Z score of net intensity
p=0.0163

Supplementary Figure 13. ALK4 loss drives galectin-3 upregulation and MGAT5-mediated glycosylation. a Western blot analysis of ALK4 and galectin-3 protein levels and qRT-PCR analysis of LGALS3 mRNA expression in MDA-MB-231 CRISPR control (NTC) and ALK4 KO cells. **b** Flow cytometric analysis of MGAT5-modified glycans in MDA-MB-231 NTC and ALK4 KO cells labeled with PHA-L lectin. The fold change in fluorescence intensity (GeoMean), normalized to NTC samples, is shown. **c** qRT-PCR analysis of MGAT5 mRNA expression in MDA-MB-231 NTC and ALK4 KO cells, with GAPDH as the reference control. **d** Fold change of UDP-GlcNAc levels in the indicated groups. **e–f** qRT-PCR analysis of mRNA expression for key enzymes involved in the hexosamine biosynthetic pathway in PANC-1 NTC cells and ALK4 KO lines (e) and MDA-MB-231 NTC and ALK4 KO lines (f). **g** Western blot analysis of GFAT2 protein levels in PANC1 and MDA-MB-231 CRISPR control (NTC) and ALK4 KO cells. **h–I** qRT-PCR analysis of mRNA expression for enzymes involved in N-branching glycan synthesis in PANC-1 NTC cells and ALK4 KO lines (h) and MDA-MB-231 NTC and ALK4 KO lines (i). **j–k** Lectin array analysis of glycan levels in PANC-1 NTC and ALK4 KO cell lysates. **j** Heatmap of lectin array results showing normalized Z-scores for all lectin stains in NTC and ALK4 KO cells. Lectin intensities with changes >2-fold and p value <0.05 are indicated by #. **k** The bar graph shows net PHA-L staining intensity in NTC and ALK4 KO groups. For in vitro analysis, each experiment was done with at least 3 independent biological replicates. For multiple comparisons with one independent variable, ordinary one-way ANOVA was used followed by Dunnett's multiple comparisons test. For comparison between two groups, data were analyzed using two-tail Student’s t tests. Data are presented as mean values +/- SEM.

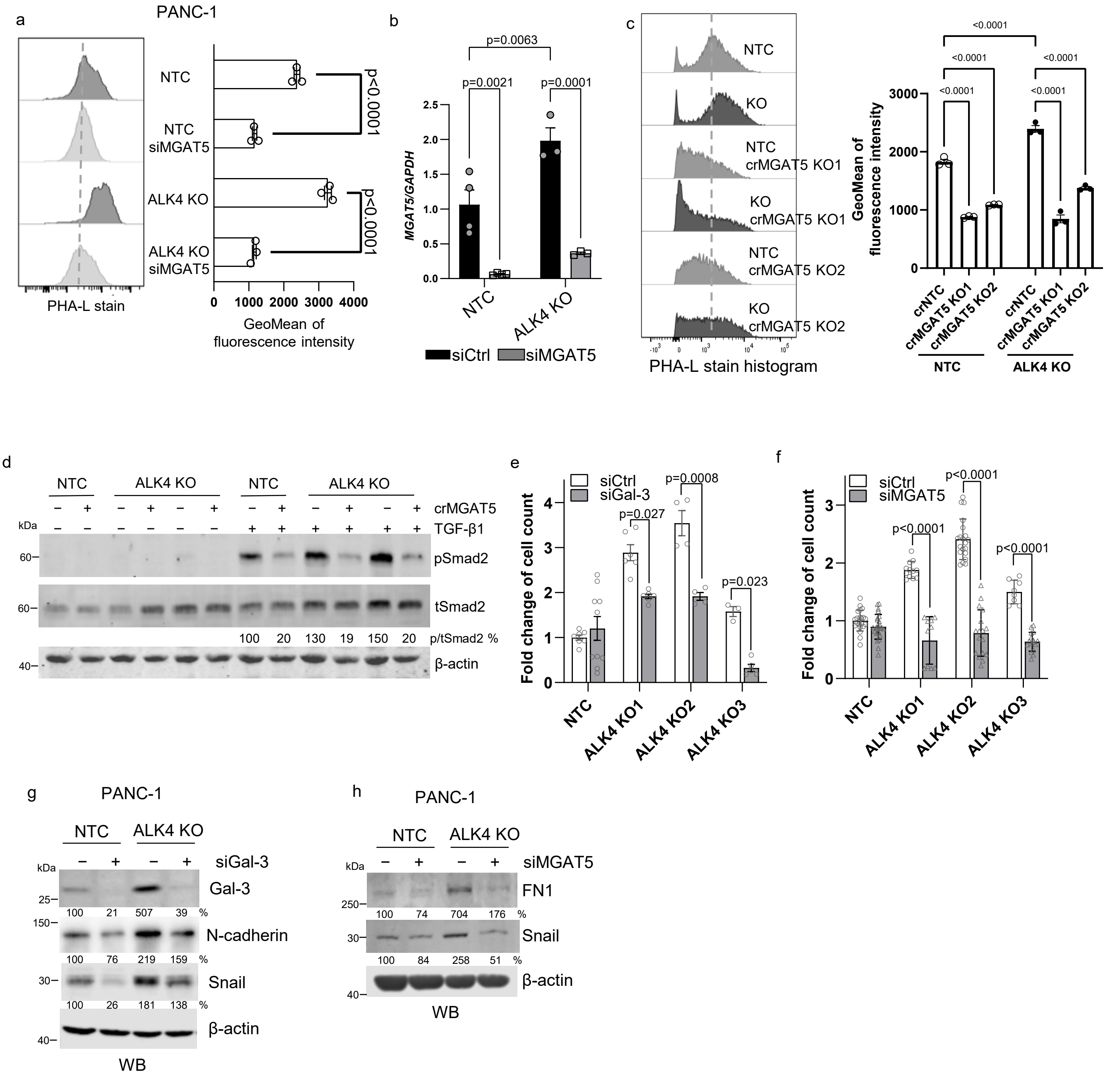

Supplementary Figure 14. MGAT5 and galectin-3 mediate ALK4 loss-induced cancer progression. **a** PANC-1 control (NTC) and ALK4 KO cells (n=3 per group) were transfected with control or MGAT5 siRNA for 3 days. MGAT5-modified glycans were detected using PHA-L lectin and analyzed by flow cytometry. Representative histograms and GeoMean fluorescence intensities are shown. **b** qRT-PCR analysis of MGAT5 mRNA levels in PANC-1 NTC and ALK4 KO cells (n=4 per group) transfected with control or MGAT5 siRNA for 3 days. GAPDH was used as the internal control. **c** Flow cytometric analysis of MGAT5-catalyzed glycans in PANC-1 NTC, NTC crMGAT5 KOs, ALK4 KO, and ALK4 KO crMGAT5 KOs cells (n=3 per group), detected with PHA-L lectin. Representative histograms and bar graphs of GeoMean fluorescence intensity. p value is provided. **d** PANC-1 NTC, NTC crMGAT5 KOs, ALK4 KO, and ALK4 KO crMGAT5 KOs cells were serum starved for 3 hours and treated with or without TGF-β1 for 30min as indicated. Lysates were used for assessing indicated proteins using Western blotting. **e-f** transwell migration assays of PANC-1 NTC and ALK4 KO cells transfected with control or (**e**) galectin-3-specific siRNA or (**f**) MGAT5-specific siRNA for 4–6 days. The migrated cells were assessed in a blinded manner. **g** Western blot analysis of galectin-3, N-cadherin, Snail, and β-actin in PANC-1 NTC and ALK4 KO cells transfected with control or galectin-3 siRNA for 4 or 5 days. **h** Western blot analysis of FN1, Snail, and β-actin in PANC-1 NTC and ALK4 KO cells transfected with control or MGAT5-specific siRNA for 4 or 5 days. Each experiment was done with at least 3 independent biological replicates. For experiments with two independent variables, data were analyzed using ordinary two-way ANOVA followed by Tukey's multiple comparisons test. Data are presented as mean values +/- SEM.

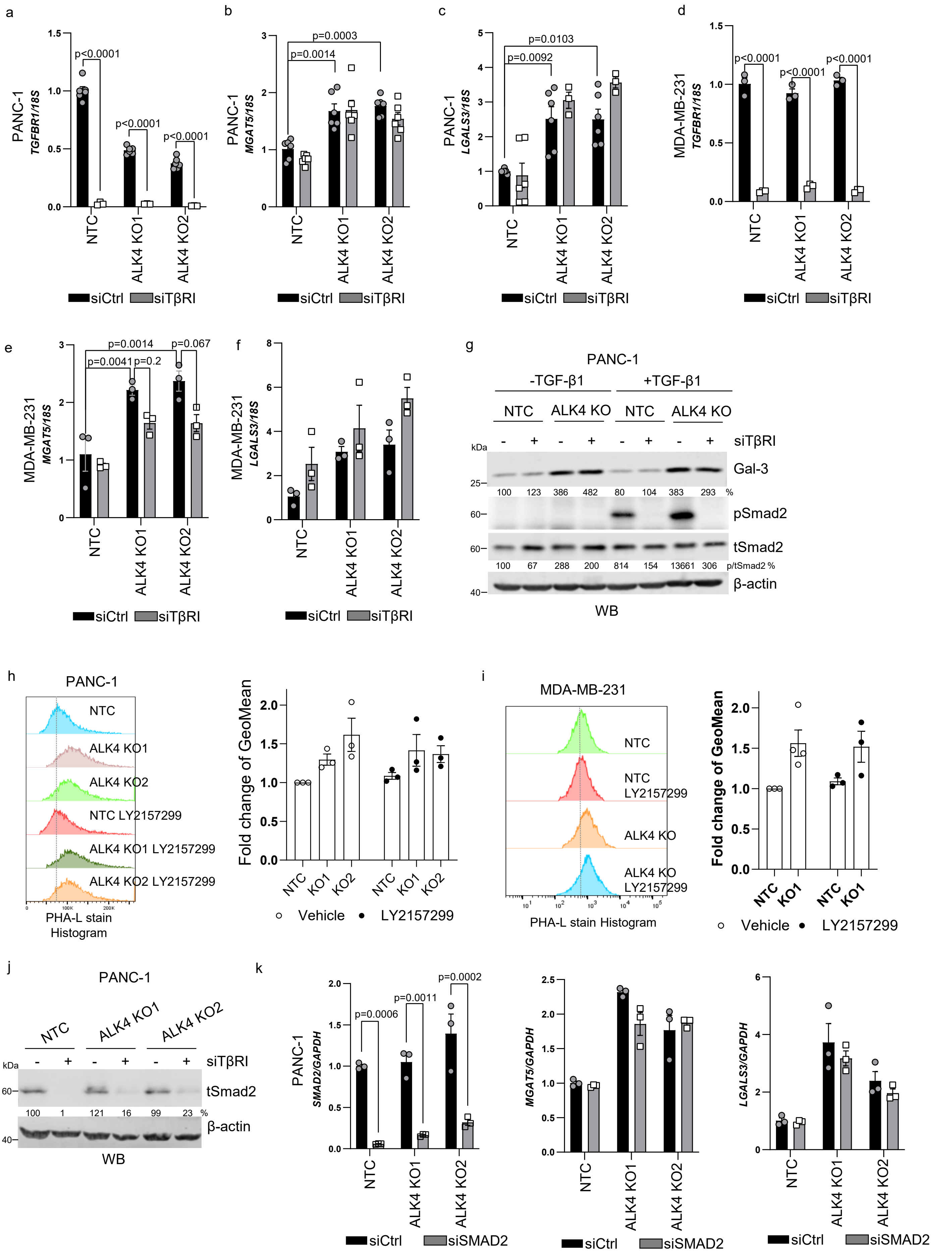
a
PANC-1 TGFBR1/18S
p<0.0001
NTC
ALK4 KO1
ALK4 KO2
siCtrl siTβRI
b
PANC-1 MGAT5/18S
p=0.0003
p=0.0014
c
PANC-1 LGALS3/18S
p=0.0103
p=0.0092
d
MDA-MB-231 TGFBR1/18S
e
MDA-MB-231 MGAT5/18S
p=0.0014
p=0.067
p=0.0041
p=0.2
f
MDA-MB-231 LGALS3/18S
g
PANC-1
-TGF-β1
+TGF-β1
NTC ALK4 KO NTC ALK4 KO
siTβRI
Gal-3
100 123 386 482 80 104 383 293 %
pSmad2
tSmad2
100 67 288 200 814 154 13661 306 p/tSmad2 %
β-actin
WB
h
PANC-1
NTC
ALK4 KO1
ALK4 KO2
NTC LY2157299
ALK4 KO1 LY2157299
ALK4 KO2 LY2157299
PHA-L stain Histogram
Fold change of GeoMean
Vehicle LY2157299
i
MDA-MB-231
NTC
NTC LY2157299
ALK4 KO
ALK4 KO LY2157299
PHA-L stain Histogram
j
PANC-1
NTC ALK4 KO1 ALK4 KO2
siTβRI
tSmad2
100 1 121 16 99 23 %
β-actin
WB
k
PANC-1 SMAD2/GAPDH
p=0.0006
p=0.0011
p=0.0002
MGAT5/GAPDH
LGALS3/GAPDH
siCtrl siSMAD2

Supplementary Figure 15. ALK4 loss promotes galectin-3 expression and MGAT5 activity through mechanisms independent of canonical TGF-β signaling. a–c qRT-PCR analysis of *TGFBR1*, *MGAT5*, and *LGALS3* mRNA levels in PANC-1 NTC and ALK4 KO cells (n=6 per group) transfected with control or TβRI siRNA for 4 days. **d–f** qRT-PCR analysis of *TGFBR1*, *MGAT5*, and *LGALS3* mRNA levels in MDA-MB-231 NTC and ALK4 KO cells (n=3 per group) transfected with control or TβRI siRNA for 4 days. **g** Western blot analysis of galectin-3, pSmad2, total Smad2, and β-actin in PANC-1 NTC and ALK4 KO cells transfected with control or TβRI siRNA for 4 days. Cells were serum-starved for 3 h and treated with TGF-β1 for 30 min before harvesting. **h–i** Flow cytometric analysis of MGAT5-catalyzed glycans labeled with PHA-L lectin in PANC1 NTC (**h**) and MDA-MB-231 NTC (**i**) and ALK4 KO cells treated with 5 μM LY2157299 for 2 days. Fold changes in GeoMean fluorescence intensities from three independent experiments are shown as bar graphs. **j** Western blot analysis of Smad2 and β-actin in PANC-1 NTC and two isogenic ALK4 KO cell lines transfected with control or SMAD2-specific siRNA for 3 days. **k** qRT-PCR analysis of *SMAD2*, *MGAT5* and *LGALS3* mRNA levels in PANC-1 NTC and ALK4 KO cell lines transfected with control or SMAD2-specific siRNA for 3 days (n=3 per group). For experiments with two independent variables, data were analyzed using ordinary two-way ANOVA followed by Tukey's multiple comparisons test. For comparison between two groups, data were analyzed using two-tail Student’s t tests. Data are presented as mean values +/- SEM.

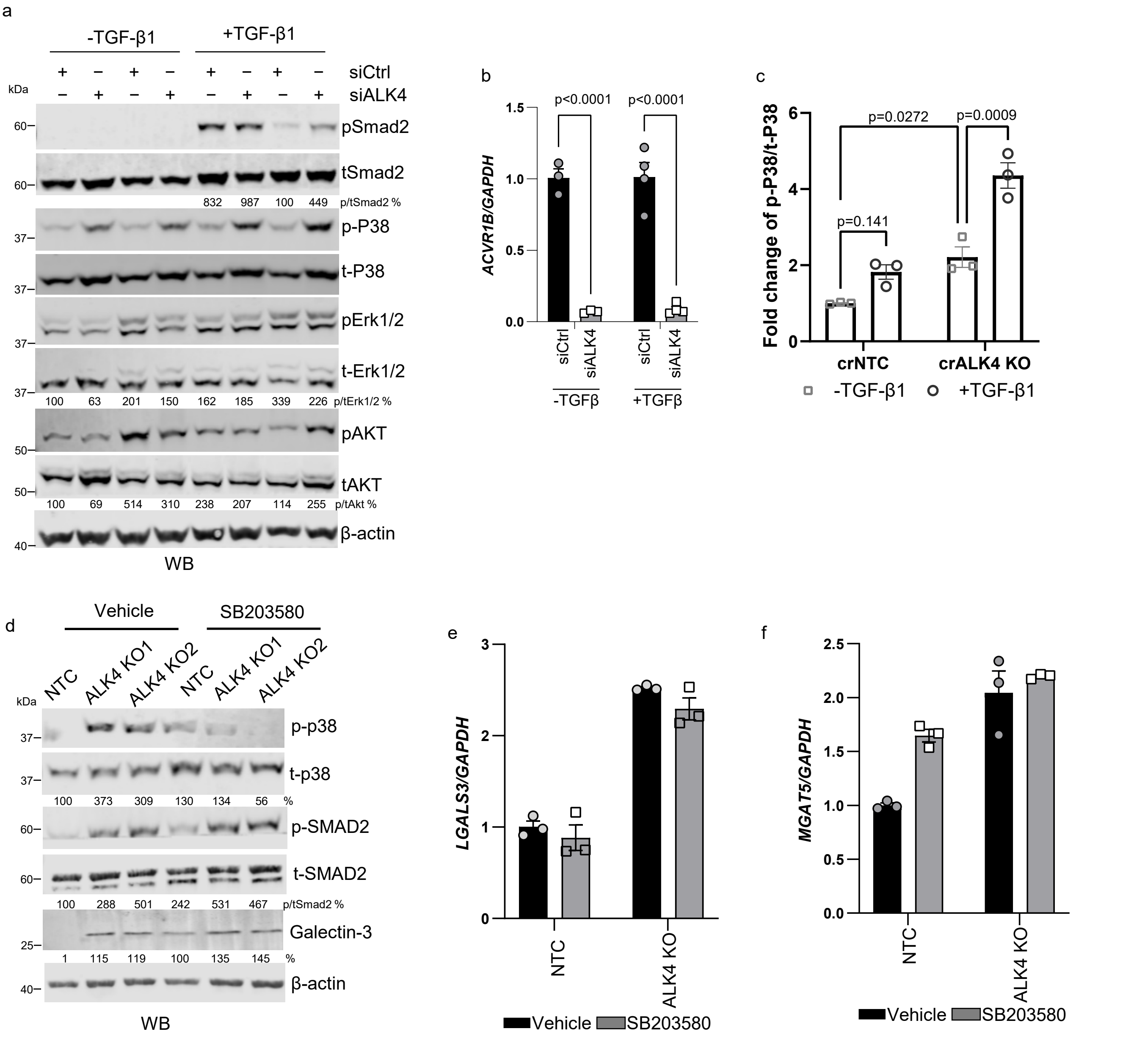

Supplementary Figure 16. ALK4 loss promotes galectin-3 expression and MGAT5 activity independently of non-canonical TGF-β signaling. a Western blot analysis of proteins involved in non-canonical TGF-β signaling. PANC-1 cells transfected with two ALK4-specific siRNAs for 72 h. Cells were serum-starved for 3 h and treated with TGF-β1 for 30 min before harvesting protein lysates. **b** qRT-PCR analysis of *ACVR1B* mRNA levels in the same samples. **c** Fold change in the p-p38/t-p38 ratio in the indicated groups, calculated from three biological and independent replicates. **d** Western blot analysis of the indicated proteins in PANC-1 NTC and two isogenic ALK4 KO cell lines treated with vehicle or 50 μM SB203580 for 3 days. Cells were serum-starved for 3 h and treated with TGF-β1 for 30 min before harvesting protein lysates. **e–f** qRT-PCR analysis of LGALS3 (**e**) and MGAT5 (**f**) mRNA levels in PANC-1 NTC and ALK4 KO cells treated with vehicle or 50 μM SB203580 for 3 days (n=3 per group). Results were repeated with 3 independent biological replicates. For experiments with two independent variables, data were analyzed using ordinary two-way ANOVA followed by Tukey's multiple comparisons test. Data are presented as mean values +/- SEM.

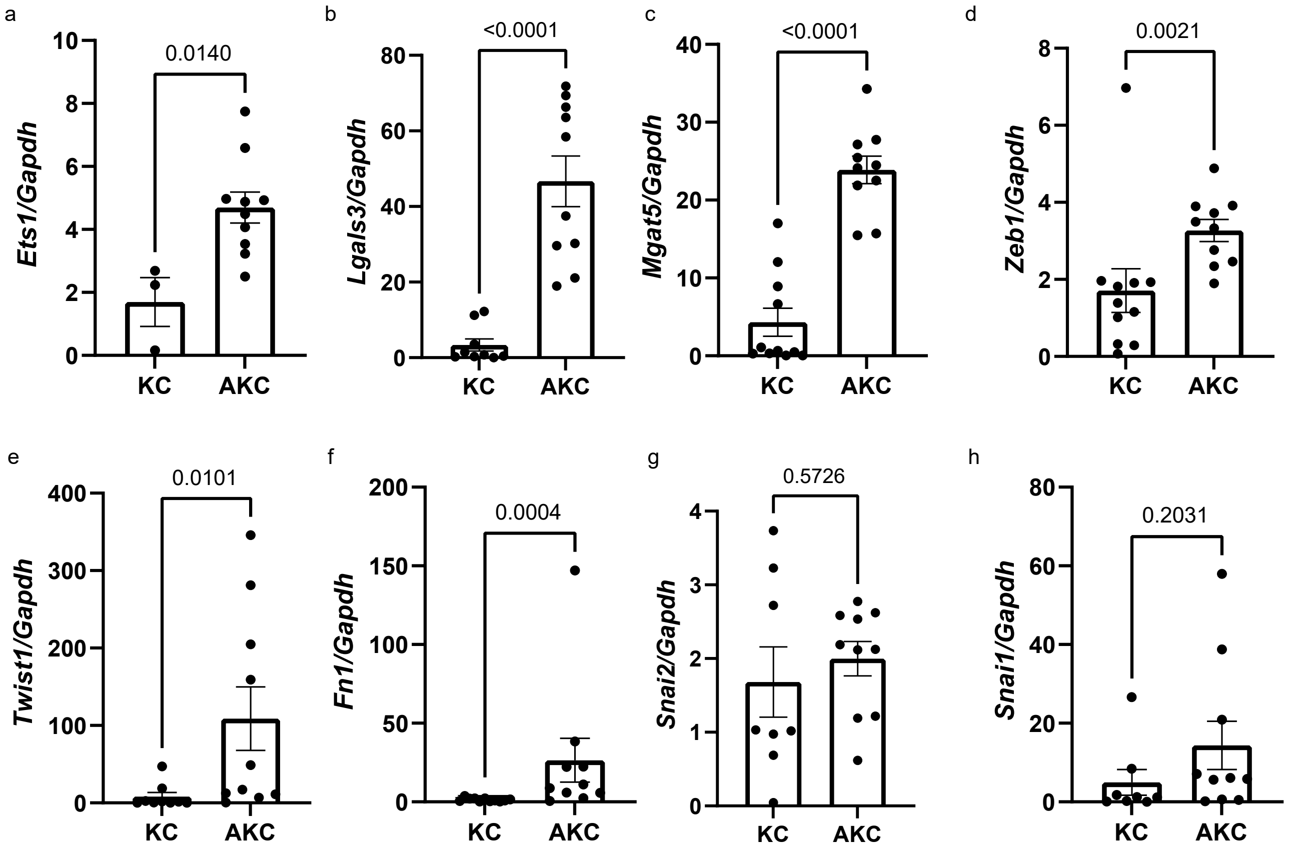

Supplementary Figure 17. Gene expression profile in pancreata from KC and AKC mice. a–h RNA was extracted from pancreatic FFPE blocks collected from AKC (n=9) and KC (n=10) mice. qRT-PCR analysis of expression of indicated genes using *Gapdh* as control. Data were analyzed using the nonparametric two-tailed Mann–Whitney test. Exact p value is provided. Data are presented as mean values +/- SEM.

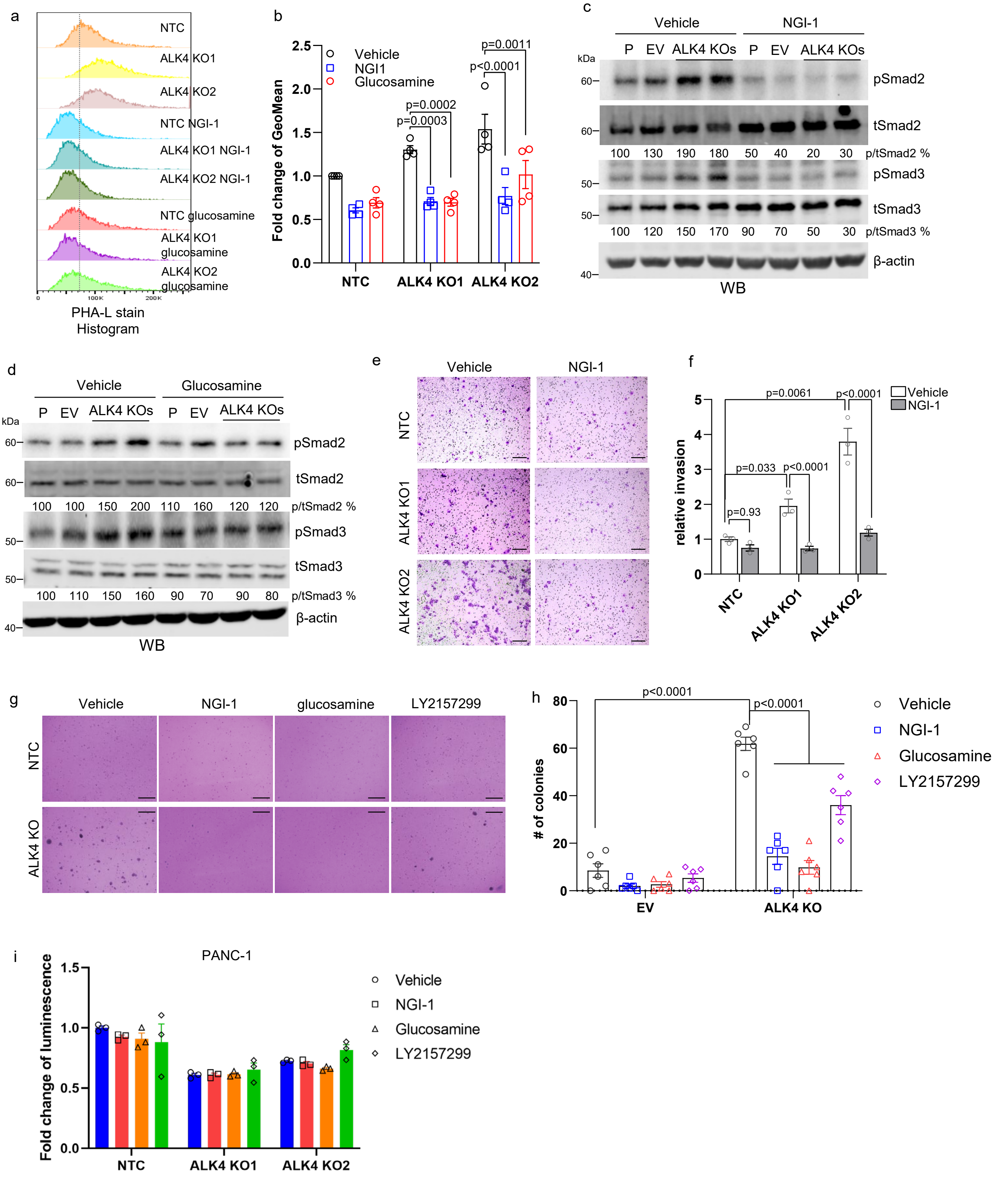
a
NTC
ALK4 KO1
ALK4 KO2
NTC NGI-1
ALK4 KO1 NGI-1
ALK4 KO2 NGI-1
NTC glucosamine
ALK4 KO1 glucosamine
ALK4 KO2 glucosamine
0
100K
200K
PHA-L stain Histogram
b
Vehicle
NGI1
Glucosamine
Fold change of GeoMean
2.5
2.0
1.5
1.0
0.5
0.0
p=0.0011
p<0.0001
p=0.0002
p=0.0003
NTC
ALK4 KO1
ALK4 KO2
c
Vehicle
NGI-1
kDa
P
EV
ALK4 KOs
P
EV
ALK4 KOs
60
60
50
50
40
pSmad2
tSmad2
100 130 190 180 50 40 20 30 p/tSmad2 %
pSmad3
tSmad3
100 120 150 170 90 70 50 30 p/tSmad3 %
β-actin
WB
d
Vehicle
Glucosamine
kDa
P
EV
ALK4 KOs
P
EV
ALK4 KOs
60
60
50
50
40
pSmad2
tSmad2
100 100 150 200 110 160 120 120 p/tSmad2 %
pSmad3
tSmad3
100 110 150 160 90 70 90 80 p/tSmad3 %
β-actin
WB
e
Vehicle
NGI-1
NTC
ALK4 KO1
ALK4 KO2
f
Vehicle
NGI-1
relative invasion
5
4
3
2
1
0
p=0.0061
p<0.0001
p=0.033
p<0.0001
p=0.93
NTC
ALK4 KO1
ALK4 KO2
g
Vehicle
NGI-1
glucosamine
LY2157299
NTC
ALK4 KO
h
# of colonies
80
60
40
20
0
p<0.0001
p<0.0001
EV
ALK4 KO
Vehicle
NGI-1
Glucosamine
LY2157299
i
PANC-1
Fold change of luminescence
1.5
1.0
0.5
0.0
Vehicle
NGI-1
Glucosamine
LY2157299
NTC
ALK4 KO1
ALK4 KO2

Supplementary Figure 18. Inhibiting N-glycosylation suppresses TGF-β signaling and cell invasiveness. a Flow cytometric analysis of MGAT5-catalized glycan levels in PANC-1 NTC and ALK4 KO cells treated with 10 μM NGI-1, 2.5 mM glucosamine, or 5 μM LY2157299 for 3 days, using PHA-L lectin staining (n=4 per group). **b** Fold change in GeoMean of PHA-L staining from (**a**). **c–d** Western blot analysis of TGF-β induced signaling proteins in PANC-1 NTC and ALK4 KO cells treated with (**c**) 10 μM NGI-1 or (**d**) 2.5 mM glucosamine for 2 days. Cells were serum-starved for 3 h and treated with 100 pM TGF-β1 for 30 min before harvesting cell lysates. **e** Transwell invasion assay of PANC-1 crNTC and crALK4 KO cells treated with DMSO or 10 μM NGI-1 for 2 days (n=3 per group). Scale bar=200 μm. **f** Fold change in cell counts from (**e**) quantitated in a blinded manner. **g-h** Soft agar colony formation assay of PANC-1 NTC and ALK4 KO cells treated with 10 μM NGI-1, 2.5 mM glucosamine, or 5 μM LY2157299 (n=6 per group). Representative images were taken at 5× magnification. Scale bar=2 mm. Colonies greater than 200 μm were counted using ImageJ in a blinded manner. **i** Viable cell number of PANC-1 crNTC and crALK4 KO cells (n=3 per group) treated with 10 μM NGI-1, 2.5 mM glucosamine, or 2.5 μM LY2157299 for 2 days, assessed using Cell TiterGlo 2.0. Data were analyzed using ordinary 2-way ANOVA followed by Tukey's multiple comparisons test. For in vitro analysis, each experiment was done with 3 independent biological replicates. Data are presented as mean values +/- SEM.

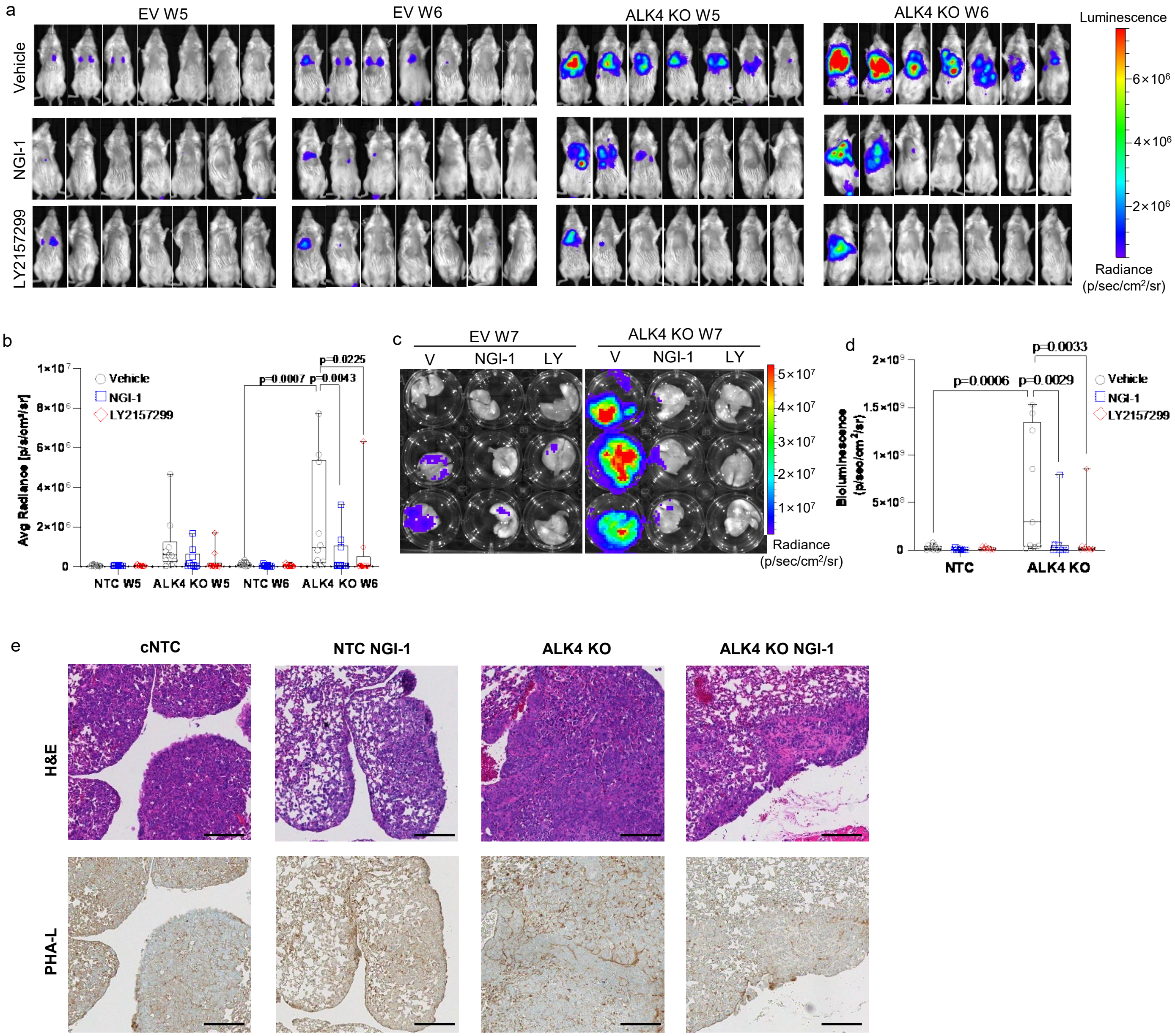

Supplemental Figure 19. Inhibiting N-glycosylation using NGI-1 or glucosamine effectively suppresses the development of pulmonary lesions. Bioluminescence imaging of pulmonary lesions in NSG mice injected via tail vein with PANC-1 crNTC and crALK4 KO cells. Mice were treated with vehicle, NGI-1, or LY2157299 (n=10 per group). **a** Representative images of live mice at weeks 5 and 6 post-injection, indicating pulmonary lesions. **b** Average radiance of pulmonary lesions from (a) at weeks 5 and 6. **c** Mice were sacrificed seven weeks after injection. Lungs were dissected, incubated with D-luciferin, and imaged. **d** Bioluminescence intensity of lung lesions at week 7. **e** Histology panel of lung sections from the indicated group stained with H&E and PHA-L lectin. Scale bar=150 μm. Data were analyzed using ordinary 2-way ANOVA followed by Tukey's multiple comparisons test. Data are presented as mean values +/- SEM.

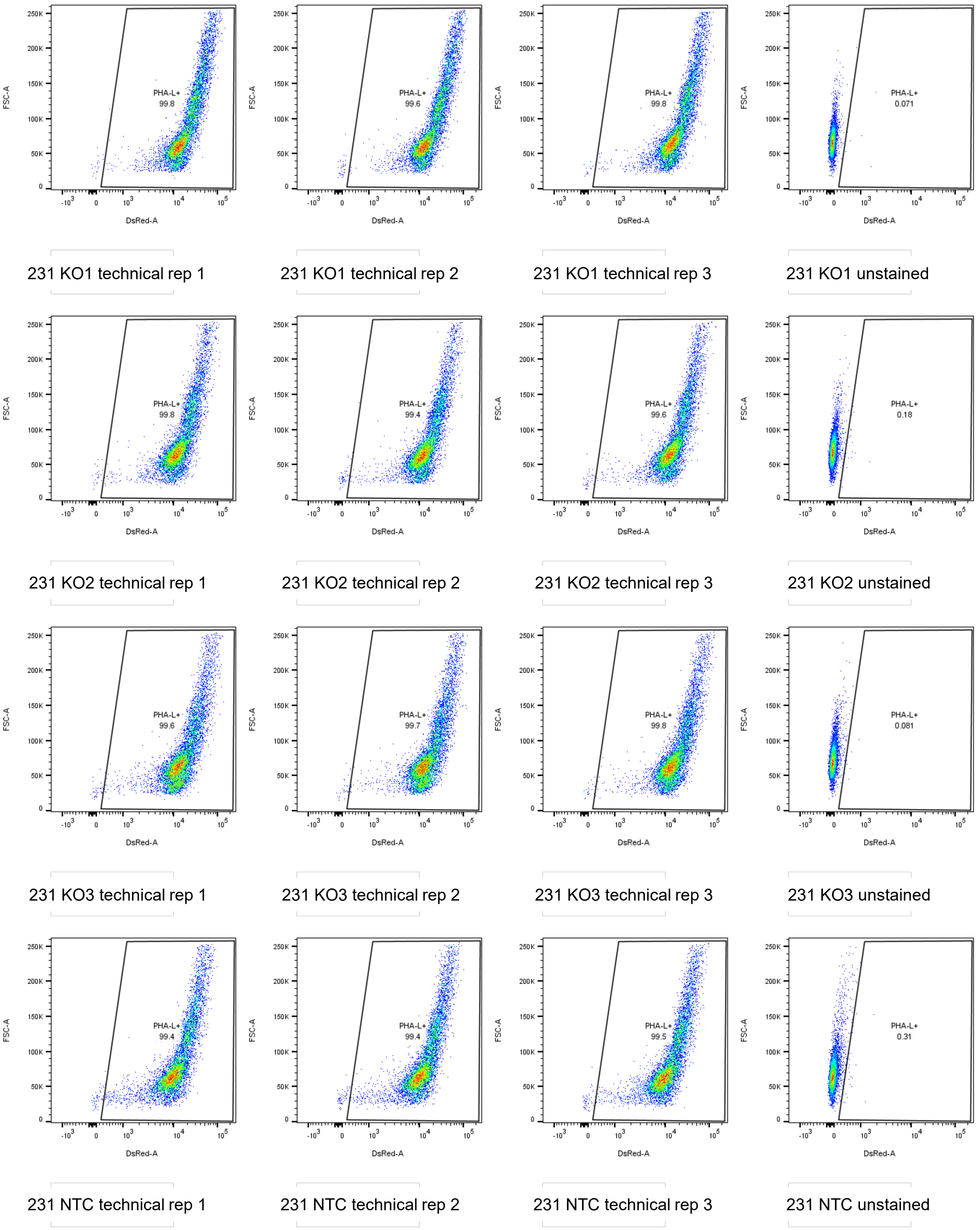

Supplemental Figure 20. Example for gating strategy (SP Fig 13b)
