# Supplementary material for: Loss of ALK4 promotes cancer progression through regulating TGF-β receptor N-glycosylation
Source: Nat Commun. 2025 Dec 17;17:854. doi: 10.1038/s41467-025-67563-1 (PMC12828005; doi:10.1038/s41467-025-67563-1)
Supplement: Supplementary file 2 — Description of Additional Supplementary Files [file 41467_2025_67563_MOESM2_ESM.pdf]

Description of Additional Supplementary Files

Supplementary Data 1: Antibodies and Sequences

Supplementary Data 2: STRING connections

Supplementary Data 3: PDAC patient summary
