# Supplementary material for: Loss of ALK4 promotes cancer progression through regulating TGF-β receptor N-glycosylation
Source: Nat Commun. 2025 Dec 17;17:854. doi: 10.1038/s41467-025-67563-1 (PMC12828005; doi:10.1038/s41467-025-67563-1)
Supplement: Supplementary file 6 — Reporting Summary [file 41467_2025_67563_MOESM6_ESM.pdf]

nature portfolio

Corresponding author(s): Gerard Blobe

Last updated by author(s): 9/27/2025

Reporting Summary

Nature Portfolio wishes to improve the reproducibility of the work that we publish. This form provides structure for consistency and transparency in reporting. For further information on Nature Portfolio policies, see our Editorial Policies and the Editorial Policy Checklist.

Statistics

For all statistical analyses, confirm that the following items are present in the figure legend, table legend, main text, or Methods section.

n/a	Confirmed	
☐	☒	The exact sample size (*n*) for each experimental group/condition, given as a discrete number and unit of measurement
☐	☒	A statement on whether measurements were taken from distinct samples or whether the same sample was measured repeatedly
☐	☒	The statistical test(s) used AND whether they are one- or two-sided<br>*Only common tests should be described solely by name; describe more complex techniques in the Methods section.*
☐	☒	A description of all covariates tested
☐	☒	A description of any assumptions or corrections, such as tests of normality and adjustment for multiple comparisons
☐	☒	A full description of the statistical parameters including central tendency (e.g. means) or other basic estimates (e.g. regression coefficient) AND variation (e.g. standard deviation) or associated estimates of uncertainty (e.g. confidence intervals)
☐	☒	For null hypothesis testing, the test statistic (e.g. *F*, *t*, *r*) with confidence intervals, effect sizes, degrees of freedom and *P* value noted<br>*Give P values as exact values whenever suitable.*
☒	☐	For Bayesian analysis, information on the choice of priors and Markov chain Monte Carlo settings
☒	☐	For hierarchical and complex designs, identification of the appropriate level for tests and full reporting of outcomes
☒	☐	Estimates of effect sizes (e.g. Cohen's *d*, Pearson's *r*), indicating how they were calculated

Our web collection on statistics for biologists contains articles on many of the points above.

Software and code

Policy information about availability of computer code

Data collection	For in vivo imaging, data were captured and analyzed using a Caliper IVIS Spectrum with Living Image Software (version 4.2); for orthographical tumor model, the tumor is measured with caliper.<br>For blots, the quantification was performed using Image Studio or Image J.<br>For microarray, the data were collected and analyzed using SensoSpot Fluorescence Microarray Analyzer.<br>For gene expression data, cycle threshold values were determined using Bio-Rad CFX Manager.
Data analysis	Data analyses related to proteomic data were performed in R/RShiny using limma/edgR2 and Quickomics (https://github.com/interactivereport/Quickomics/). DEPs and DEGs were analyzed using DAVID BIOINFORMATICS and/or GSEA with default setting. For cell migration/invasion/colony formation assay, the samples were analyzed using Image J. For flow cytomytry experiments, data were analyzed using FlowJo v10. All other statistical analysis was conducted with GraphPad Prism v9.0.0.

For manuscripts utilizing custom algorithms or software that are central to the research but not yet described in published literature, software must be made available to editors and reviewers. We strongly encourage code deposition in a community repository (e.g. GitHub). See the Nature Portfolio guidelines for submitting code & software for further information.

Data

Policy information about availability of data

All manuscripts must include a data availability statement. This statement should provide the following information, where applicable:

- Accession codes, unique identifiers, or web links for publicly available datasets
- A description of any restrictions on data availability
- For clinical datasets or third party data, please ensure that the statement adheres to our policy

The patient and transcriptomic data were acquired from cBioPortal (https://www.cbioportal.org/datasets) or NCBI Gene Expression Omnibus (GEO) repository. Raw data of proteomic analysis has been deposited in MassIVE with accession number MSV000091598 (https://massive.ucsd.edu/ProteoSAFe/dataset.jsp?task=699b8d9fa544472f8856ae14bf823ce6). The author declare that all data generated or analyzed during this study are available upon reasonable request.

Research involving human participants, their data, or biological material

Policy information about studies with human participants or human data. See also policy information about sex, gender (identity/presentation), and sexual orientation and race, ethnicity and racism.

Reporting on sex and gender	N/A
Reporting on race, ethnicity, or other socially relevant groupings	N/A
Population characteristics	N/A
Recruitment	N/A
Ethics oversight	*Identify the organization(s) that approved the study protocol.*

Note that full information on the approval of the study protocol must also be provided in the manuscript.

Field-specific reporting

Please select the one below that is the best fit for your research. If you are not sure, read the appropriate sections before making your selection.

☒ Life sciences ☐ Behavioural & social sciences ☐ Ecological, evolutionary & environmental sciences

For a reference copy of the document with all sections, see nature.com/documents/nr-reporting-summary-flat.pdf

Life sciences study design

All studies must disclose on these points even when the disclosure is negative.

Sample size	For in vivo tumor study, we performed a power analysis to calculate the number of animals needed to reach statistical significance. Using our prior papers as a guide, we have determined that our proposed studies require a sample size of 7-8 mice per group, as this will give us a 90% power to detect a difference in means of 15% between control and experimental groups with a standard deviation of 10% (Power = 0.90; alpha = 0.05). In this manuscript, for each animal experiment, 8-15 of mice were used per group as indicated in figure legends. For in vitro analysis, at least 3 independent and biological replicates were done.
Data exclusions	No data were excluded from the analysis
Replication	Number of replicates is described in the figure legends. All attempts of replication were successful. Animal experiments were done with injection of multiple cell lines, either gain or loss of function models, and spaced more than one year apart for each to ensure reproducibility of the phenotypes.
Randomization	In all in vitro or in vivo studies, individuals/samples were randomly assigned for treatment or injections.
Blinding	The investigators were blinded to group allocation during data collection and analysis whenever applicable.

Reporting for specific materials, systems and methods

We require information from authors about some types of materials, experimental systems and methods used in many studies. Here, indicate whether each material, system or method listed is relevant to your study. If you are not sure if a list item applies to your research, read the appropriate section before selecting a response.

Materials & experimental systems

n/a	Involved in the study
	☒ Antibodies
	☒ Eukaryotic cell lines
☒	☐ Palaeontology and archaeology
	☒ Animals and other organisms
☒	☐ Clinical data
☒	☐ Dual use research of concern
☒	☐ Plants

Methods

n/a	Involved in the study
☒	☐ ChIP-seq
	☒ Flow cytometry
☒	☐ MRI-based neuroimaging

Antibodies

Antibodies used

ZO-1 (CST, #5406); β-catenin (CST, #8480), FN1 (CST, #26836); E-cadherin (CST, #14472); N-cadherin (CST, #13116); Vimentin (CST, #5741); α-SMA (CST, #19245); pSmad2 (CST, #18338); tSmad2 (CST, #3103); pSmad3 (CST #9520); tSmad3 (CST, #9513); β-actin (CST, #3700); ALK4 (R&D Systems, AF222); TβRI (R&D Systems, AF3025); TβRII (R&D Systems, AF-241-NA); Galectin-3 (CST, 12733); CK19 (CST, #12434); Zeb1 (CST, #70512); Slug (CST, #9585); Snail (CST, #3879); pSmad2 (CST, #3108); Smad4 (CST, 46535); phospho-P38 (CST, 4511); total p38 (CST, 9212); phospho-AKT (CST, 9271); total AKT (CST, 4691); Ki67(CST, 9449), phospho-Erk1/2 (CST, 9101), total-Erk1/2 (CST, 9102), Ki67 (CST, 9449), GFAT2(Proteintech, B-1115-2)

Validation

ZO-1 (CST, #5406):detect both mouse and human ZO-1, has 69 citations on CST website (https://www.cellsignal.com/products/primary-antibodies/zo-1-antibody/5406)
β-catenin (CST, #8480): this antibody has been validated using SimpleChIP® Enzymatic Chromatin IP Kits (https://www.cellsignal.com/products/primary-antibodies/b-catenin-d10a8-xp-rabbit-mab/8480)
FN1 (CST, #26836): there are 124 citations on CST website (https://www.cellsignal.com/products/primary-antibodies/fibronectin-fn1-e5h6x-rabbit-mab/26836)
E-cadherin (CST, #14472): validation performed by IP (https://www.cellsignal.com/products/primary-antibodies/e-cadherin-4a2-mouse-mab/14472)
N-cadherin (CST, #13116): Monoclonal antibody is produced by immunizing animals with a synthetic peptide corresponding to residues surrounding Arg526 of human N-cadherin protein; there are 1617 citation of this antibody used in WB, IP, IF, IHC (https://www.cellsignal.com/products/primary-antibodies/n-cadherin-d4r1h-xp-rabbit-mab/13116).
Vimentin (CST, #5741): there are 3392 citations of this antibody used in WB, F, IHC, IF. This is a monoclonal antibody is produced by immunizing animals with a synthetic peptide corresponding to residues surrounding Arg45 of human vimentin protein (https://www.cellsignal.com/products/primary-antibodies/vimentin-d21h3-xp-rabbit-mab/5741)
α-SMA (CST, #19245): antibody validated by IP on the CST website (https://www.cellsignal.com/products/primary-antibodies/a-smooth-muscle-actin-d4k9n-xp-rabbit-mab/19245)
pSmad2 (CST, #18338): this antibody has been validated using SimpleChIP® Enzymatic Chromatin IP Kits (https://www.cellsignal.com/products/primary-antibodies/phospho-smad2-ser465-ser467-e8f3r-rabbit-mab/18338)
tSmad2 (CST, #3103): This is validated by siRNA knockdown (https://www.cellsignal.com/products/primary-antibodies/smad2-l16d3-mouse-mab/3103).
pSmad3 (CST, #9520): This antibody has been validated using SimpleChIP® Enzymatic Chromatin IP Kits (https://www.cellsignal.com/products/primary-antibodies/phospho-smad3-ser423-425-c25a9-rabbit-mab/9520).
tSmad3 (CST, #9513): Polyclonal antibodies are produced by immunizing animals with a synthetic peptide corresponding to a central region unique to human SMAD3. There are 223 citations of this antibody on Cell Signaling website (https://www.cellsignal.com/products/primary-antibodies/smad3-antibody/9513).
β-actin (CST, #3700): https://www.cellsignal.com/products/primary-antibodies/b-actin-8h10d10-mouse-mab/3700
ALK4 (R&D Systems, AF222): This antibody is knockout validated.
TβRI (R&D Systems, AF3025): this is a polyclonal antibody produced by immunizing animals with sequence Leu34-Glu125 of human (https://www.rndsystems.com/products/human-tgf-beta-ri-alk-5-antibody_af3025)
TβRI (Accession # P36897): t can be used in WB, ELISA
TβRII (R&D Systems, AF-241-NA):this is a polyclonal antibody produced by immunizing animals with sequence Ile24-Asp159 of human (https://www.rndsystems.com/products/human-tgf-beta-rii-antibody_af-241-na)
TβRII (Accession # P37173.2). It can be used in WB, IF, F.
Galectin-3 (CST, 12733): validated by siRNA knockdown (https://www.cellsignal.com/products/primary-antibodies/galectin-3-lgals3-antibody/12733).
CK19 (CST, #12434): Monoclonal antibody is produced by immunizing animals with a synthetic peptide corresponding to amino acids near the amino terminus of human keratin 17 and human keratin 19 proteins; there are 21 citation of this product (https://www.cellsignal.com/products/primary-antibodies/keratin-17-19-d4g2-xp-rabbit-mab/12434).
Zeb1 (CST, #70512): Monoclonal antibody is produced by immunizing animals with recombinant protein corresponding to the central region of human ZEB1 protein with 82 citations (https://www.cellsignal.com/products/primary-antibodies/zeb1-e2g6y-xp-rabbit-mab/70512).
Slug (CST, #9585): Monoclonal antibody is produced by immunizing animals with a recombinant human Slug protein with 1124 citations (https://www.cellsignal.com/products/primary-antibodies/slug-c19g7-rabbit-mab/9585).
Snail (CST, #3879):Monoclonal antibody is produced by immunizing animals with a recombinant human Snail protein with 1638 citations (https://www.cellsignal.com/products/primary-antibodies/snail-c15d3-rabbit-mab/3879).
pSmad2 (CST, #3108): Monoclonal antibody is produced by immunizing animals with a synthetic phosphopeptide corresponding to residues surrounding Ser465/467 of human SMAD2 with 1080 citations (https://www.cellsignal.com/products/primary-antibodies/phospho-smad2-ser465-467-138d4-rabbit-mab/3108).

	Smad4 (CST, #46535): Monoclonal antibody is produced by immunizing animals with a recombinant human Smad4 protein with 118 citations (https://www.cellsignal.com/products/primary-antibodies/smad4-d3r4n-xp-rabbit-mab/46535).<br>phospho-P38 (CST, #4511): Monoclonal antibody is produced by immunizing animals with a synthetic phosphopeptide corresponding to residues surrounding Thr180/Tyr182 of human p38 MAPK. protein with 3928 citations (https://www.cellsignal.com/products/primary-antibodies/phospho-p38-mapk-thr180-tyr182-d3f9-xp-rabbit-mab/4511).<br>total p38 (CST, #9212): Polyclonal antibody is produced by immunizing animals with a synthetic peptide corresponding to the sequence of human p38 MAPK with 4445 citations (https://www.cellsignal.com/products/primary-antibodies/p38-mapk-antibody/9212).<br>phospho-AKT (CST, #9271): Monoclonal antibody is produced by immunizing animals with a synthetic phosphopeptide corresponding to residues surrounding Ser473 of mouse Akt. protein with 8278 citations (https://www.cellsignal.com/products/primary-antibodies/phospho-akt-ser473-antibody/9271).<br>total AKT (CST, #4691): Monoclonal antibody is produced by immunizing animals with a synthetic peptide corresponding to residues in the carboxy-terminal sequence of mouse Akt with 5953 citations (https://www.cellsignal.com/products/primary-antibodies/akt-pan-c67e7-rabbit-mab/4691).<br>Ki67(CST, #9449): Monoclonal antibody is produced by immunizing animals with a synthetic peptide corresponding to residues near the amino terminus of human Ki-67 protein with 817 citations (https://www.cellsignal.com/products/primary-antibodies/ki-67-8d5-mouse-mab/9449).<br>GFAT2 (Proteintech, 15189-1-AP): GFPT2 fusion protein Ag7275 (330-682aa encoded by BC000012) (https://www.ptglab.com/products/GFPT2-Antibody-15189-1-AP.htm?srsltid=AfmBOooR0kBUwNHWlUjHkpuRowBAPUoVSvOz2XSlzV6B-_V9SW8kglFf)<br>Phospho-Erk1/2 (CST, #9101): Polyclonal antibodies are produced by immunizing animals with a synthetic phosphopeptide corresponding to residues surrounding Thr202/Tyr204 of human p44 MAP kinase with 9293 citations (https://www.cellsignal.com/products/primary-antibodies/phospho-p44-42-mapk-erk1-2-thr202-tyr204-antibody/9101).<br>Total-Erk1/2 (CST, #9102): Polyclonal antibodies are produced by immunizing animals with a synthetic peptide corresponding to a sequence in the C-terminus of rat p44 MAP Kinase, with 8330 citations (https://www.cellsignal.com/products/primary-antibodies/p44-42-mapk-erk1-2-antibody/9102).

Eukaryotic cell lines

Policy information about cell lines and Sex and Gender in Research

Cell line source(s)	MDA-MB-231: purchased from ATCC; human female; LM2 cells: provided by Dr. Joan Massagué (Memorial Sloan Kettering Cancer Institute); metastatic derives from MDA-MB-231 cells; MCF10A: provided by Dr. William Schiemann (Case Western Reserve University); human female; 4T1 cells: provided by Dr. William Schiemann (Case Western Reserve University); mouse cell line; PANC-1: purchased from ATCC; human male; HPNE: purchased from ATCC; human male
Authentication	Cell line authentication for cell lines used in this study were performed by Duke University DNA Analysis Facility. Briefly, DNA profiling entails analyzing DNA samples for polymorphic short tandem repeat (STR) markers. We use the GenePrint 10 kit from Promega. With this kit, the repeat regions of ten short tandem repeat loci and the Amelogenin gender marker are co-amplified and the allele sizes/identity are determined on an ABI 3130xl automated capillary DNA sequencer.
Mycoplasma contamination	Cell lines tested negative for mycoplasma contamination.
Commonly misidentified lines<br>(See ICLAC register)	No commonly misidentified lines were used.

Animals and other research organisms

Policy information about studies involving animals; ARRIVE guidelines recommended for reporting animal research, and Sex and Gender in Research

Laboratory animals	For GEM model, B6.FVB-Tg(Pdx1-cre)6Tuv/J (JAX:014647) was purchased from Jackson Laboratories (6-8 weeks), the LSL-KrasG12D; Pdx-cre1 mice were acquired from Dr. David Kirsh (Duke University), and the ALK4flox/flox mice were acquire from Dr. Philippe Bertolino (Cancer Research Centre of Lyon). The GEM mice KrasLSL-G12D;Acvr1bfl/fl;Pdx1-Cre (AKC) mice and KrasLSL-G12D;Pdx1-Cre (KC) controls were housed till 8-9 month.<br>For xenograft model, C57BL/6J mice (JAX#000664) and NU/J(JAX#002019) and NSG (JAX:005557) were purchased from Jackson Laboratories 6-8 weeks months of age.
Wild animals	N/A
Reporting on sex	Equal amount of male and female mice were used for study of pancreatic cancer models; Only female mice were used for breast cancer models.
Field-collected samples	N/A
Ethics oversight	All experiments involving animals were performed in accordance with standard protocols approved by the Duke Institutional Animal Care and Use Committee.

Note that full information on the approval of the study protocol must also be provided in the manuscript.

Plants

Seed stocks

Report on the source of all seed stocks or other plant material used. If applicable, state the seed stock centre and catalogue number. If plant specimens were collected from the field, describe the collection location, date and sampling procedures.

Novel plant genotypes

Describe the methods by which all novel plant genotypes were produced. This includes those generated by transgenic approaches, gene editing, chemical/radiation-based mutagenesis and hybridization. For transgenic lines, describe the transformation method, the number of independent lines analyzed and the generation upon which experiments were performed. For gene-edited lines, describe the editor used, the endogenous sequence targeted for editing, the targeting guide RNA sequence (if applicable) and how the editor was applied.

Authentication

Describe any authentication procedures for each seed stock used or novel genotype generated. Describe any experiments used to assess the effect of a mutation and, where applicable, how potential secondary effects (e.g. second site T-DNA insertions, mosiacism, off-target gene editing) were examined.

Flow Cytometry

Plots

Confirm that:

- [x] The axis labels state the marker and fluorochrome used (e.g. CD4-FITC).
- [x] The axis scales are clearly visible. Include numbers along axes only for bottom left plot of group (a 'group' is an analysis of identical markers).
- [ ] All plots are contour plots with outliers or pseudocolor plots.
- [ ] A numerical value for number of cells or percentage (with statistics) is provided.

Methodology

Sample preparation	PANC-1 and MDA-MB-231 cells were harvested with 0.05% Trypsin and resuspended in FACS buffer (0.1% BSA in PBS). 350,000 cells per sample were incubated in 1 ml FACS buffer with 2.5 µg/ml fluorescein conjugated PHA-L (Phaseolus Vulgaris Leucoagglutinin, Vector Laboratories, Cat# FL-1111-2). Samples were rotated in a head to end manner at 4 °C for 30 minutes in the dark. After incubation, samples were pelleted by centrifugation and then resuspended in FACS buffer for analysis.
Instrument	BD FACSCanto™ II (BD Biosciences)
Software	FlowJo™ v10 (BD Biosciences)
Cell population abundance	n/a
Gating strategy	Forward and side scatter gating (FSC-A vs SSC-A) was done to include more than 85% of the cells. Forward scatter height versus forward scatter area density plot (FSC-A vs FSC-H) was used for doublet exclusion. In this manuscript, the flow cytometry was performed with single staining of PHA-L. The single parameter histograms and scattered plot (fluorescence intensity vs SSC-A) were used for setting up boundary between positive stain vs negative stain. Using unstained samples stain intensity as cutoff, we gated positive stained cells from the selected singlets for statistic analysis.

- [x] Tick this box to confirm that a figure exemplifying the gating strategy is provided in the Supplementary Information.
