# Supplementary material for: Loss of ALK4 promotes cancer progression through regulating TGF-β receptor N-glycosylation
Source: Nat Commun. 2025 Dec 17;17:854. doi: 10.1038/s41467-025-67563-1 (PMC12828005; doi:10.1038/s41467-025-67563-1)

Fig 3A

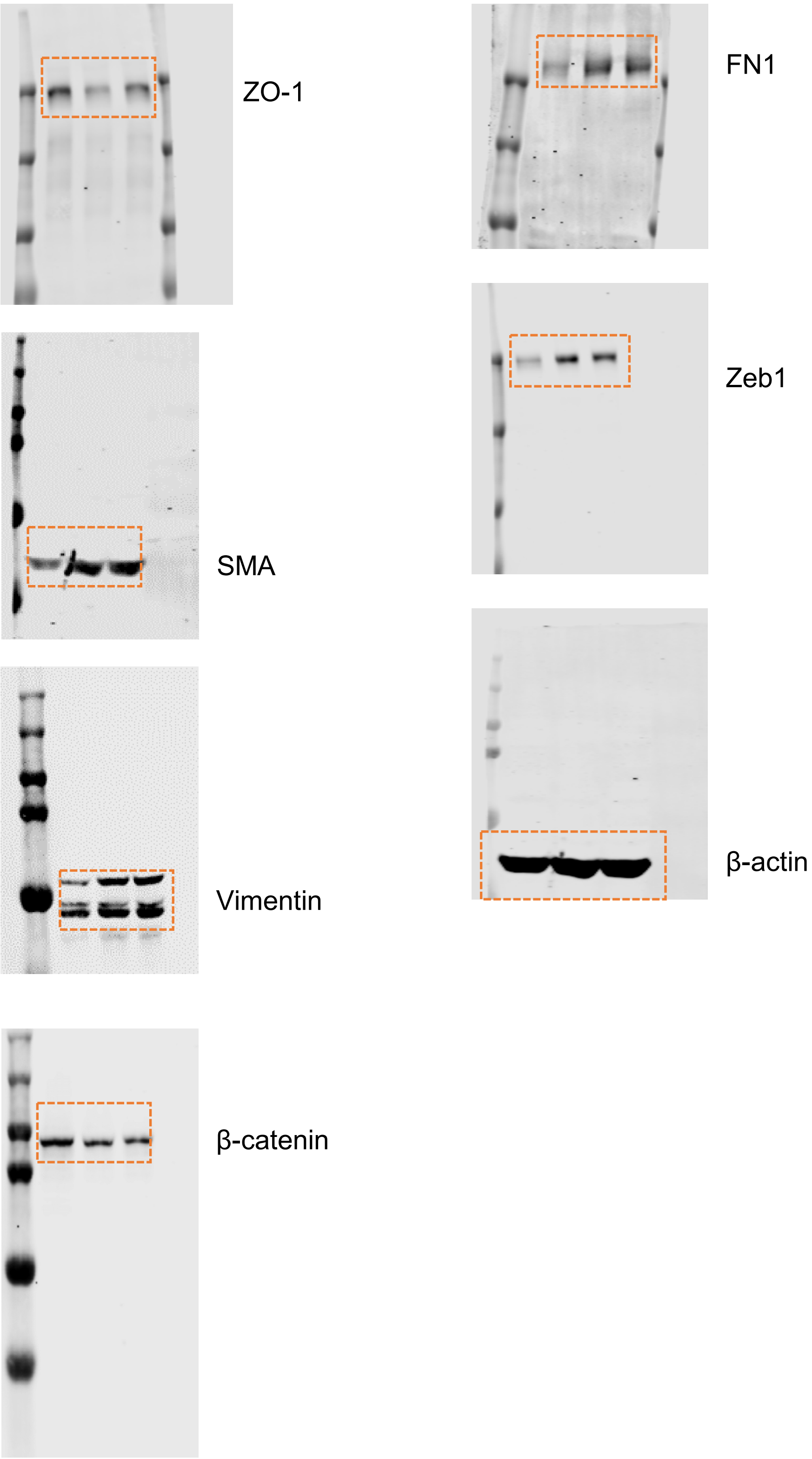

Fig 3B

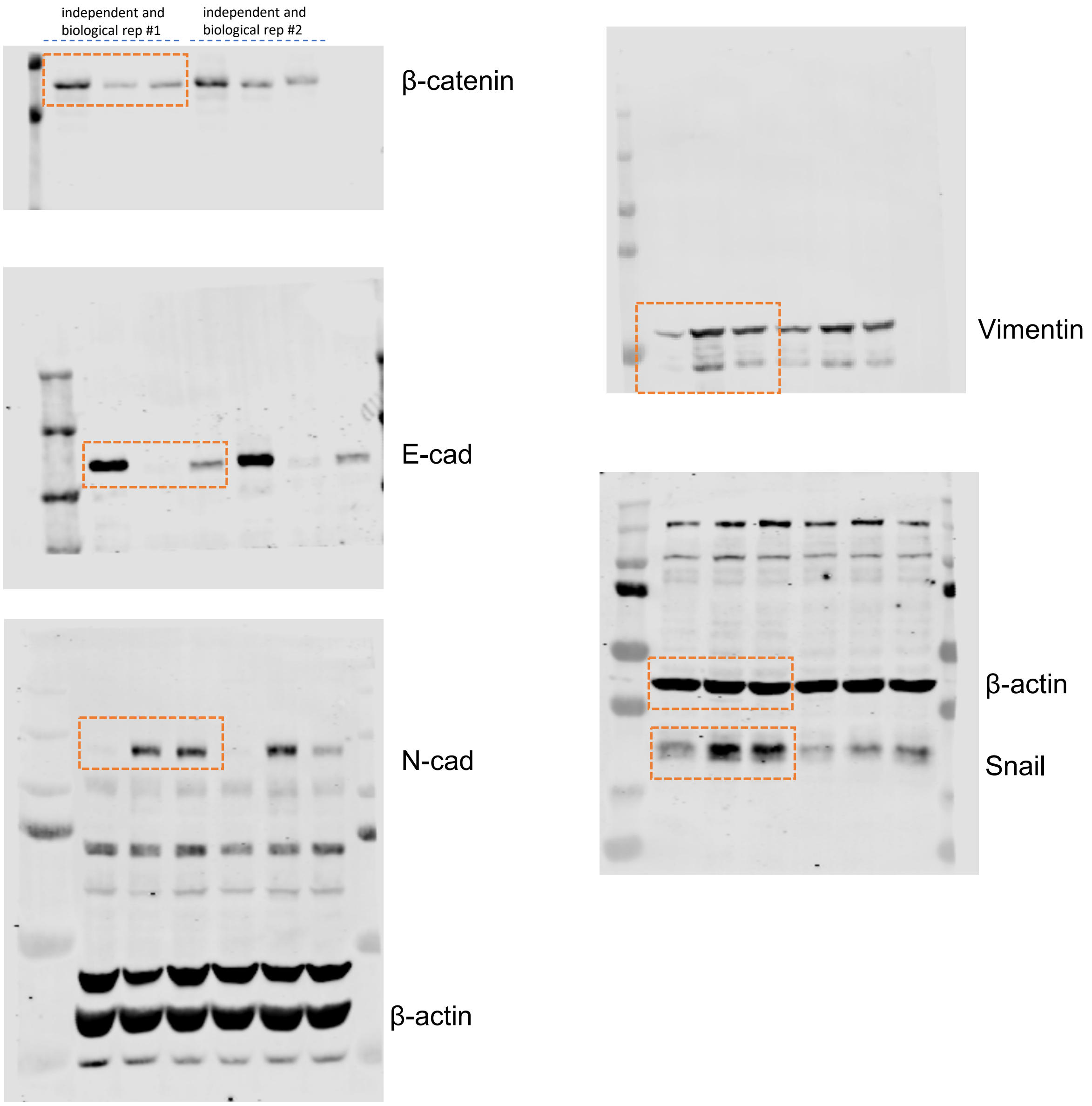
independent and biological rep #1
independent and biological rep #2
β-catenin
E-cad
N-cad
β-actin
Vimentin
β-actin
Snail

Fig 4A

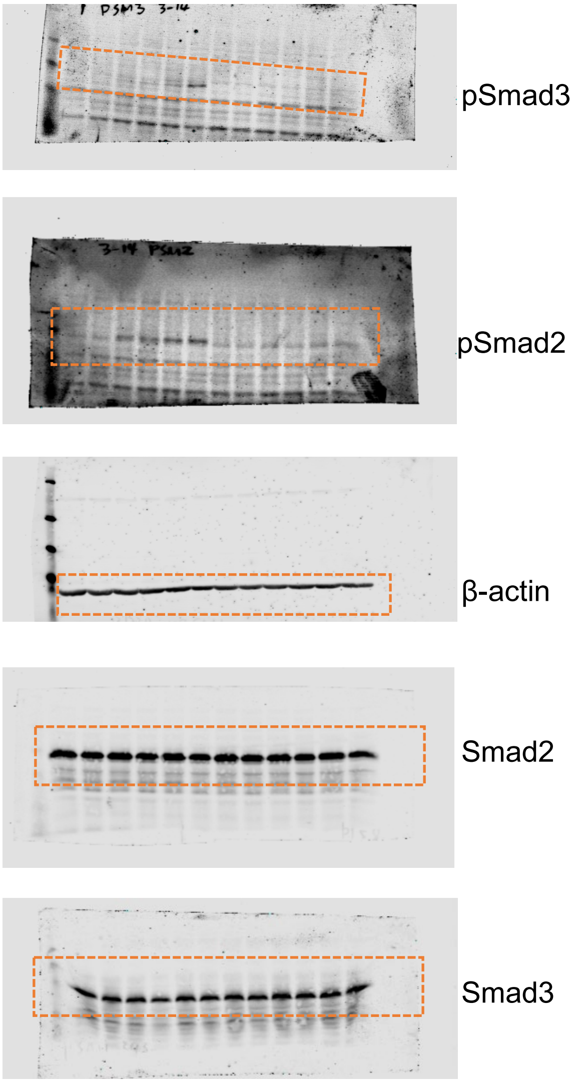
pSmad3
pSmad2
β-actin
Smad2
Smad3

Fig 4B

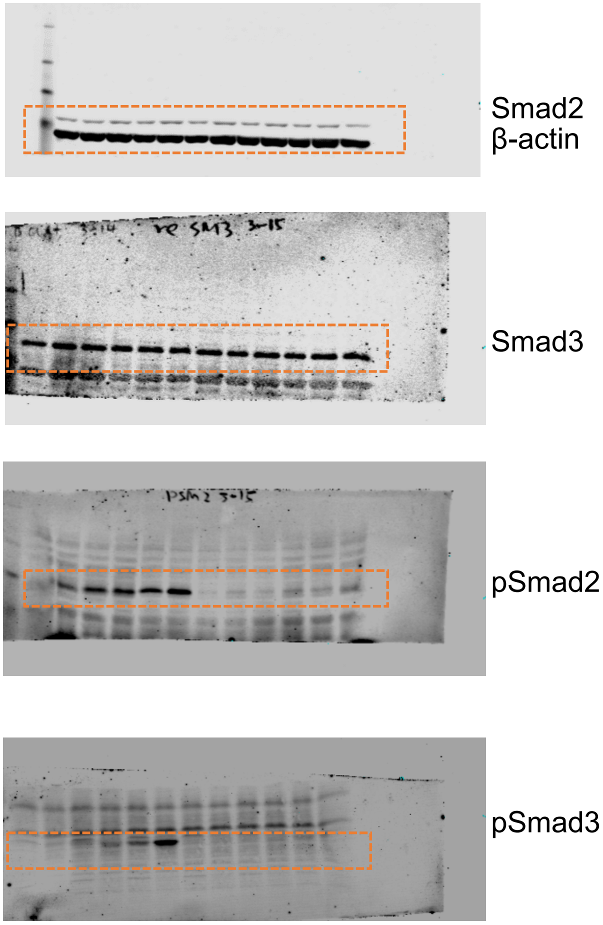
Smad2
β-actin
Smad3
pSmad2
pSmad3

Fig 4C

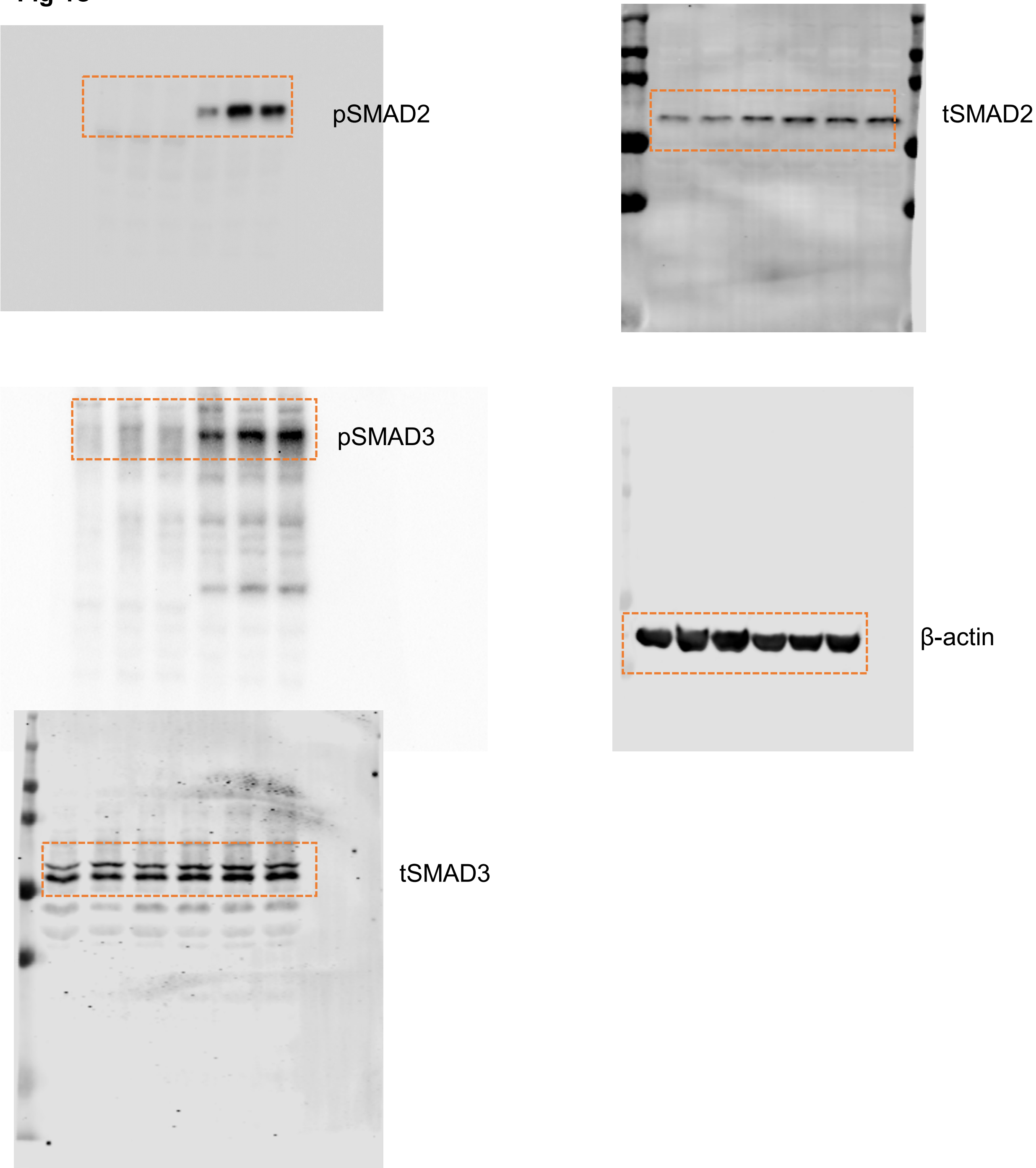

Fig 4H

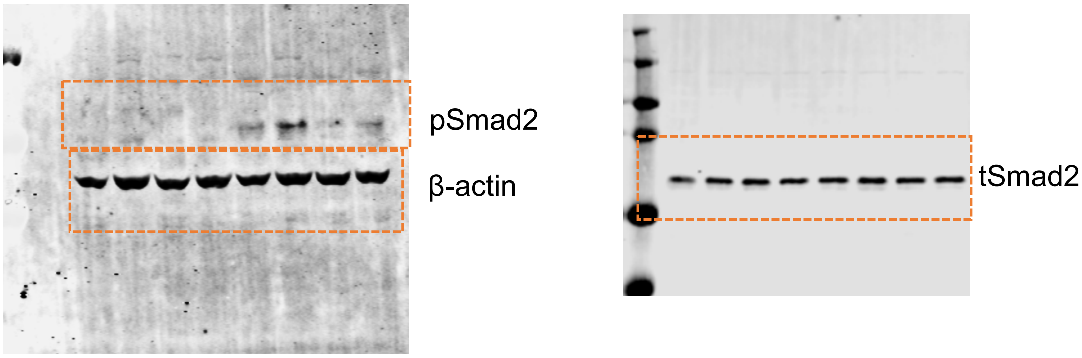

Figure 5A

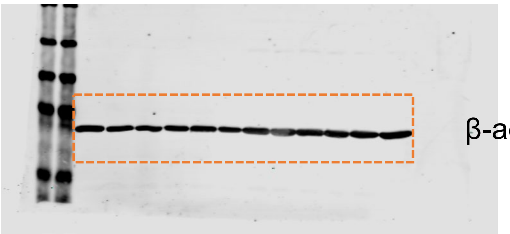

β-actin

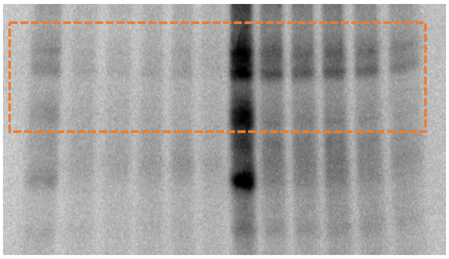

TβRI/II (binding and cross-linking assay)

Figure 5B

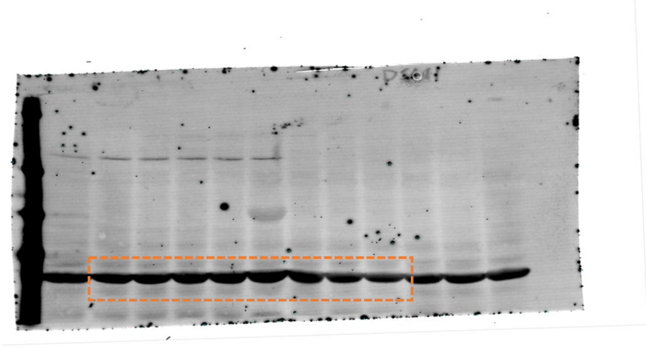

β-actin

PANC-1 shNTC Growth media no serum 4h as control

TGF-β1 treat for mins

PANC-1 shNTC 0
PANC-1 shNTC 15
PANC-1 shNTC 30
PANC-1 shNTC 60
PANC-1 shALK4 0
PANC-1 shALK4 15
PANC-1 shALK4 30
PANC-1 shALK4 60
PANC-1 Parental cells in growth media
PANC-1 shNTC in growth media
PANC-1 shALK4 growth media

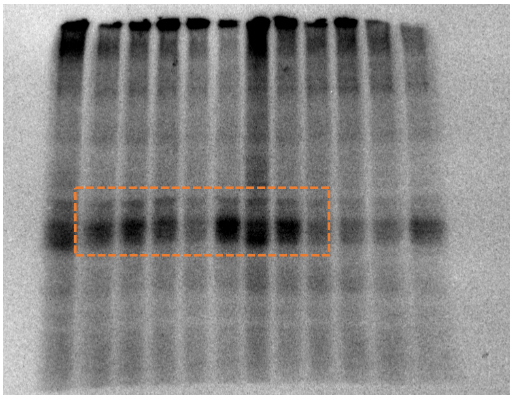

TβRI/II (binding and cross-linking assay)

Fig 5C

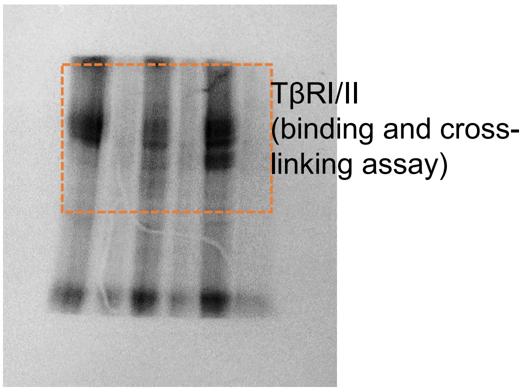

TβRI/II (binding and cross-linking assay)

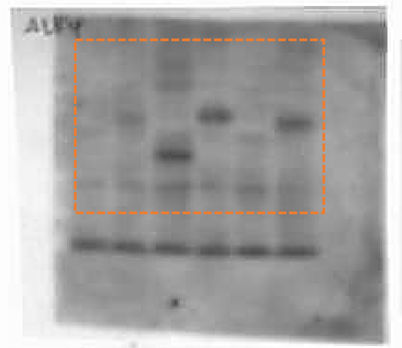

ALK4

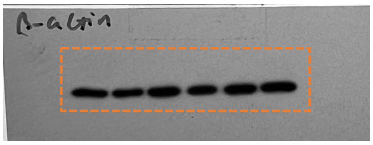

β-actin

Fig 5D

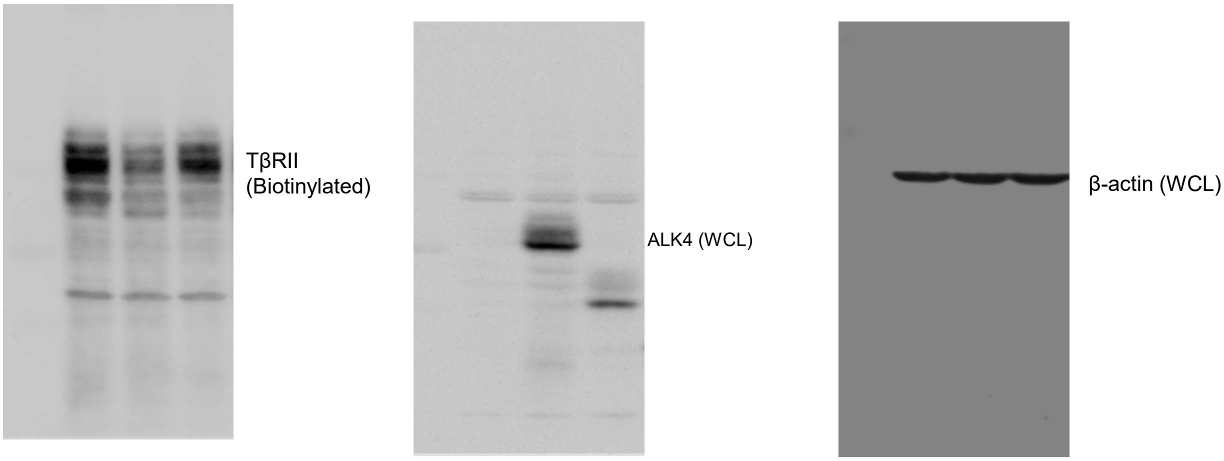

Fig 5E

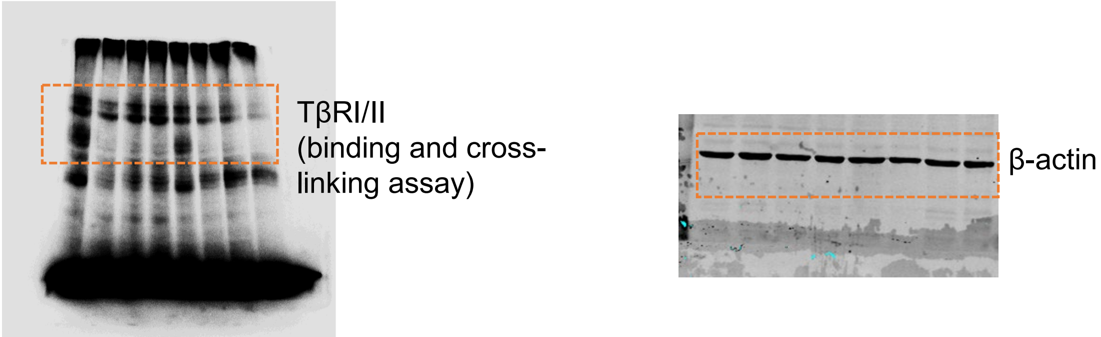

Fig 5F

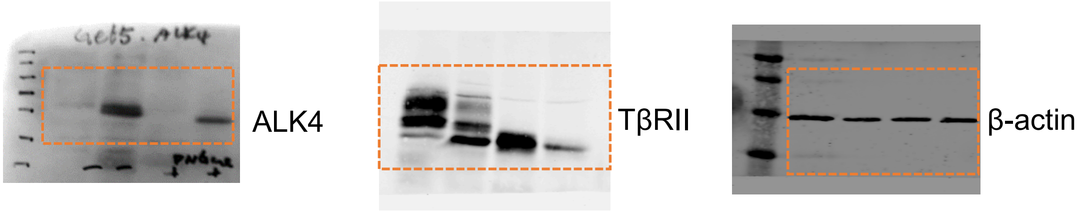

Fig 5G

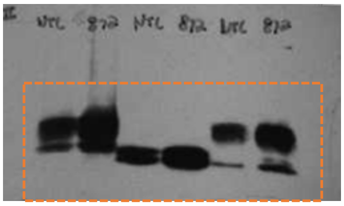

PANC-1
TβRII

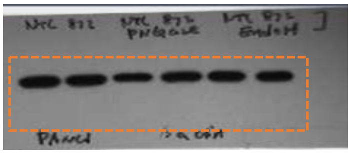

PANC-1
β-actin

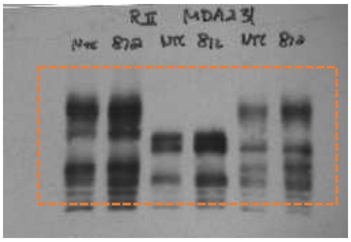

MDA MB 231
RII

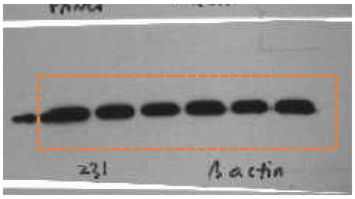

MDA MB 231
β-actin

Figure 5H

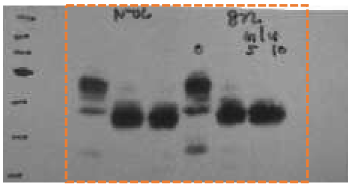

TβRII

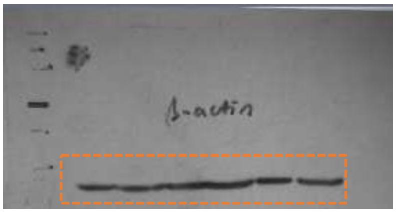

β-actin

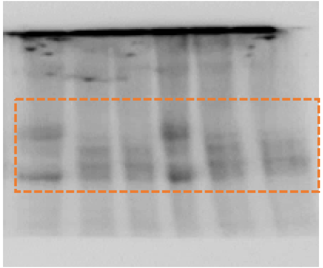

TβRI/II
(binding and cross-
linking assay)

Figure 5I

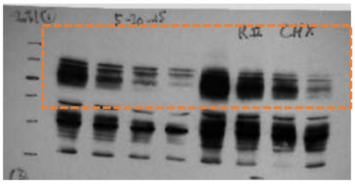

TβRII

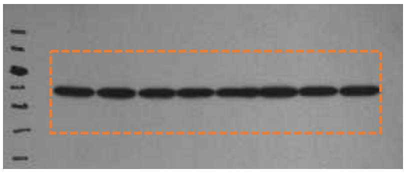

β-actin

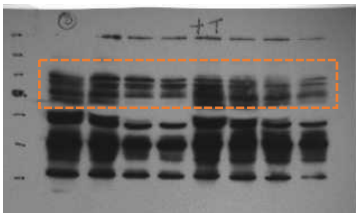

TβRII

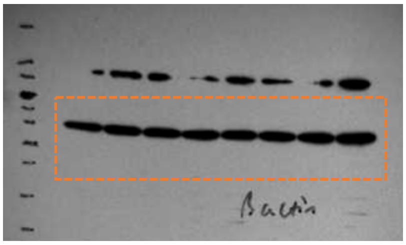

β-actin

Figure 7A

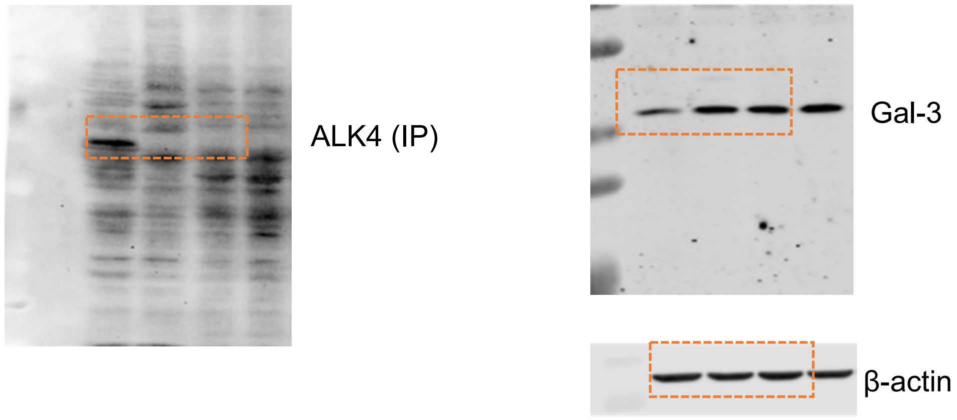

Figure 7D

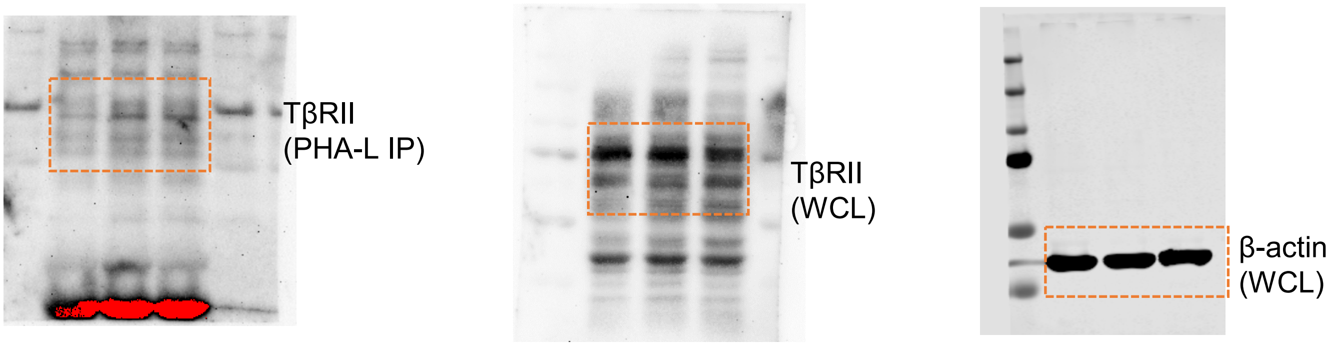

Figure 7E

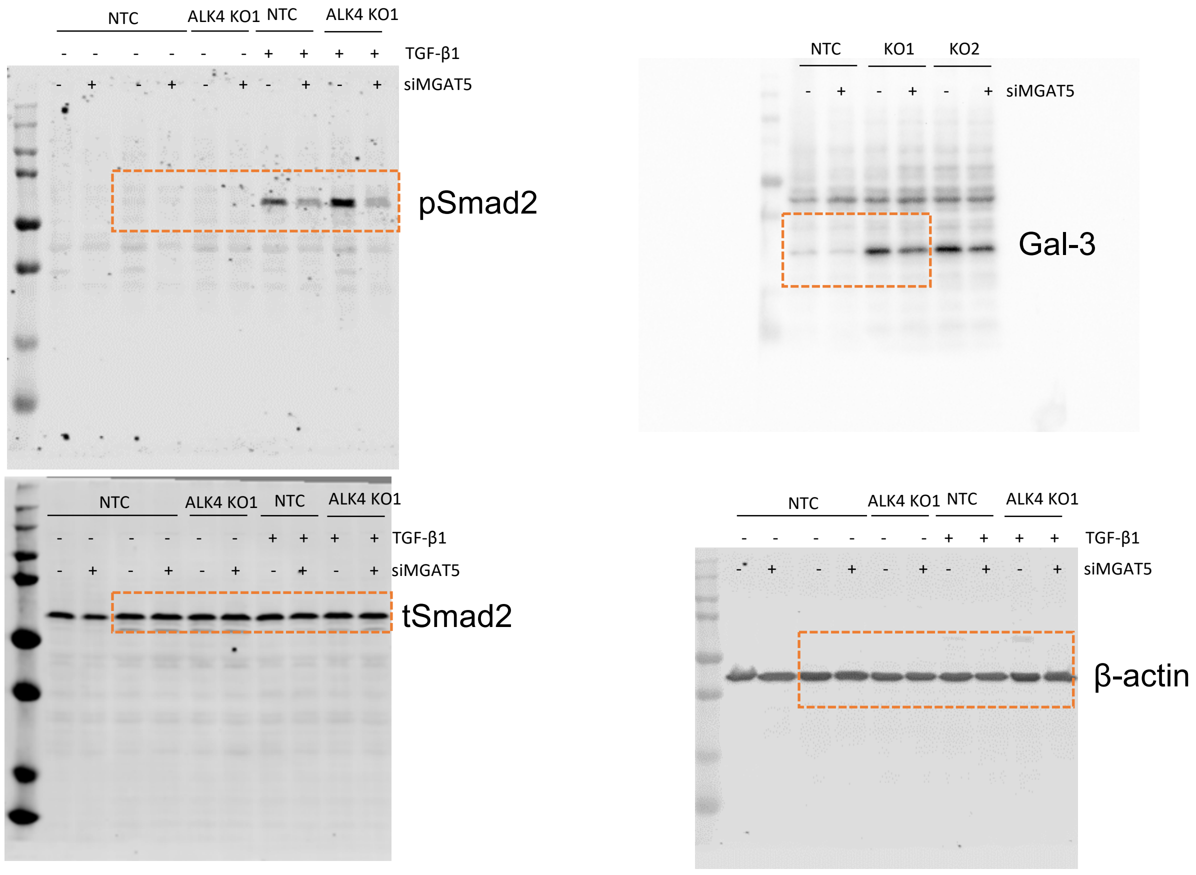

Figure 7F

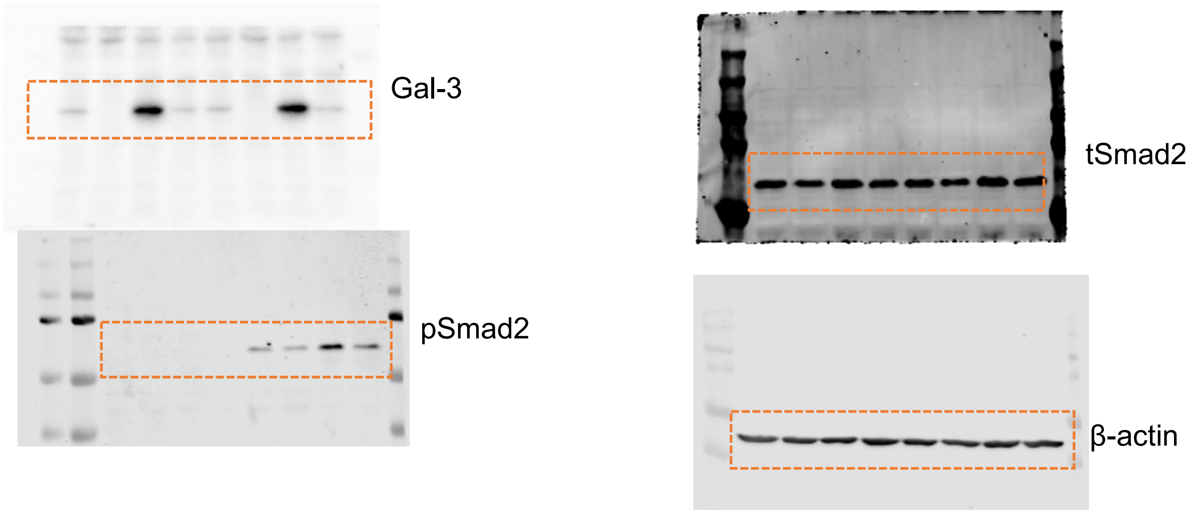

Figure 7G

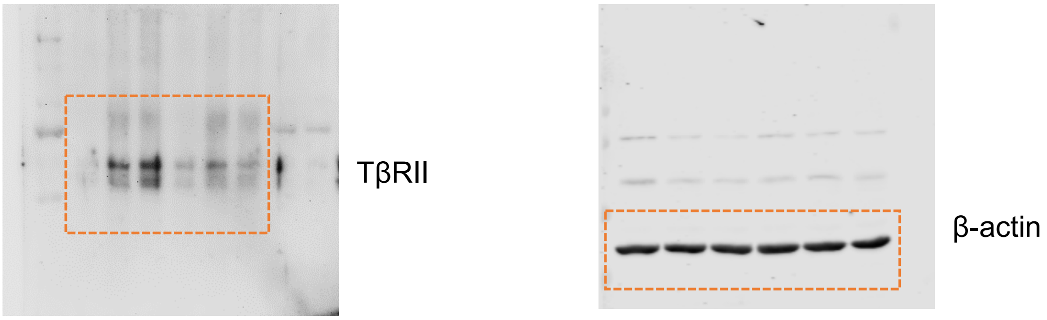

SP Fig 3A

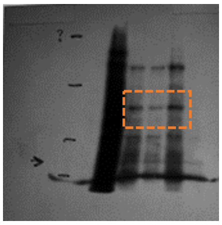

ALK4 (IP)

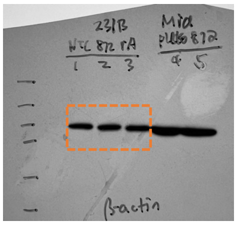

β-actin

SP Fig 3B

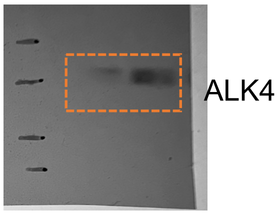

ALK4

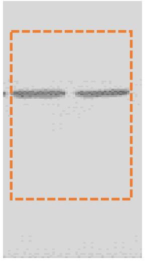

β-actin

SP Fig 3C

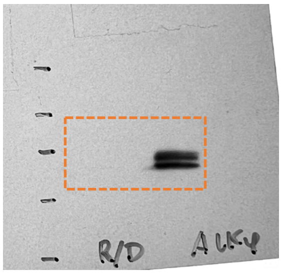

ALK4

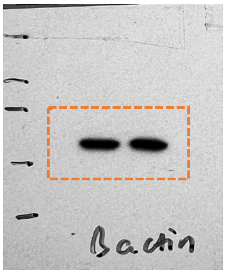

β-actin

SP Fig 3J

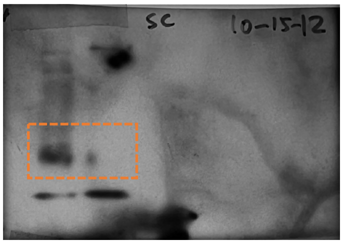

ALK4 (IP)

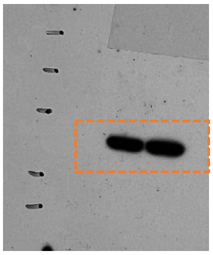

β-actin

SP Fig 5A

50 kDa–

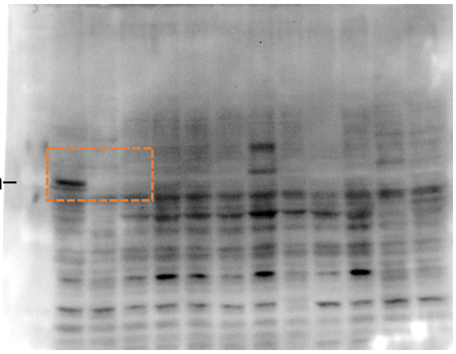

ALK4 (IP)

50 kDa–

37 kDa–

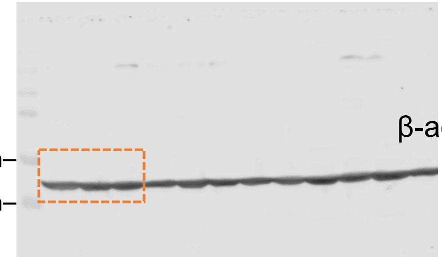

β-actin

SP Fig 5B

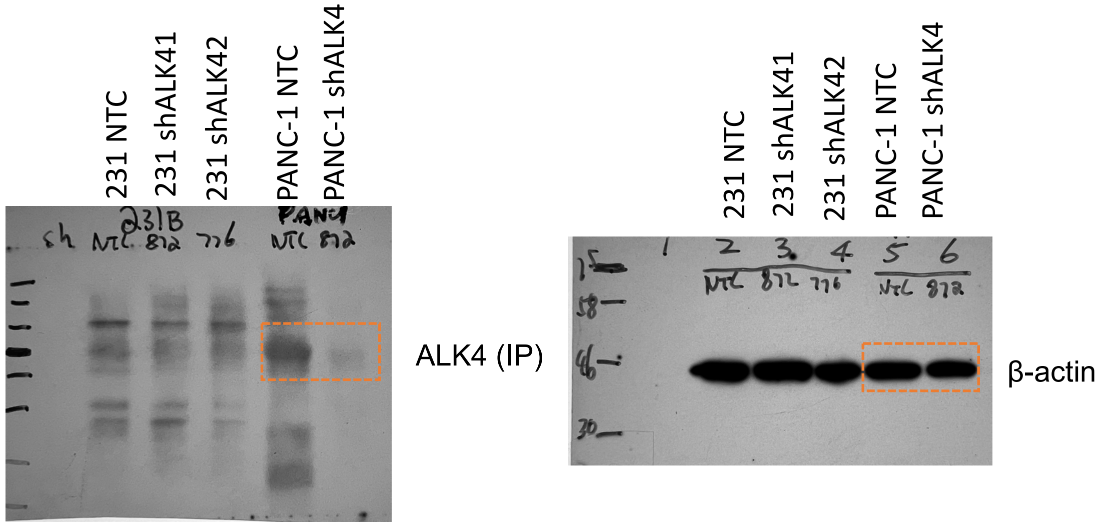

SP Fig 5H

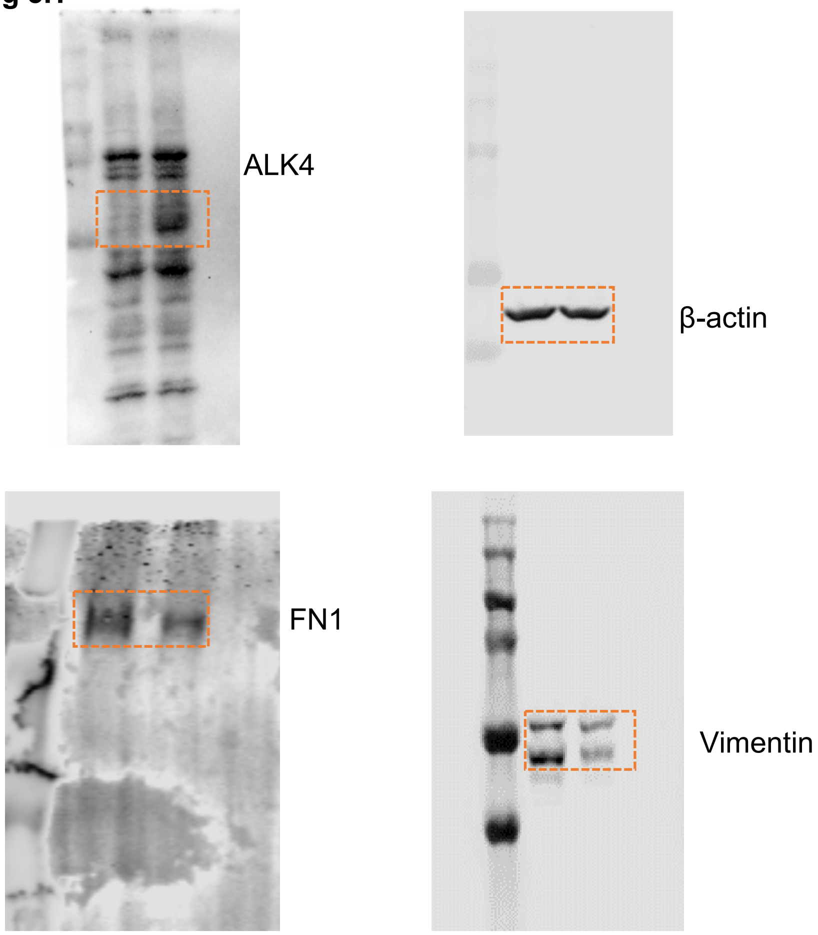

SP Fig 8C

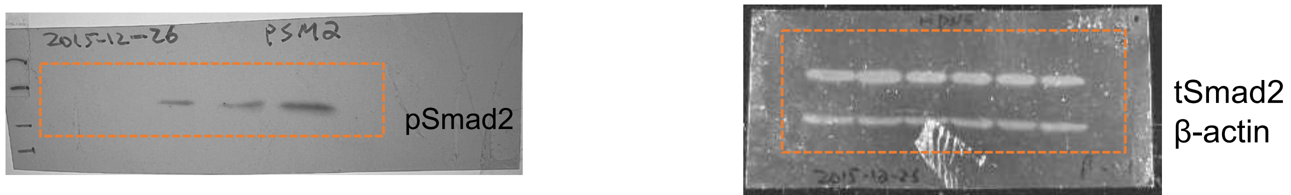

SP Fig 9A

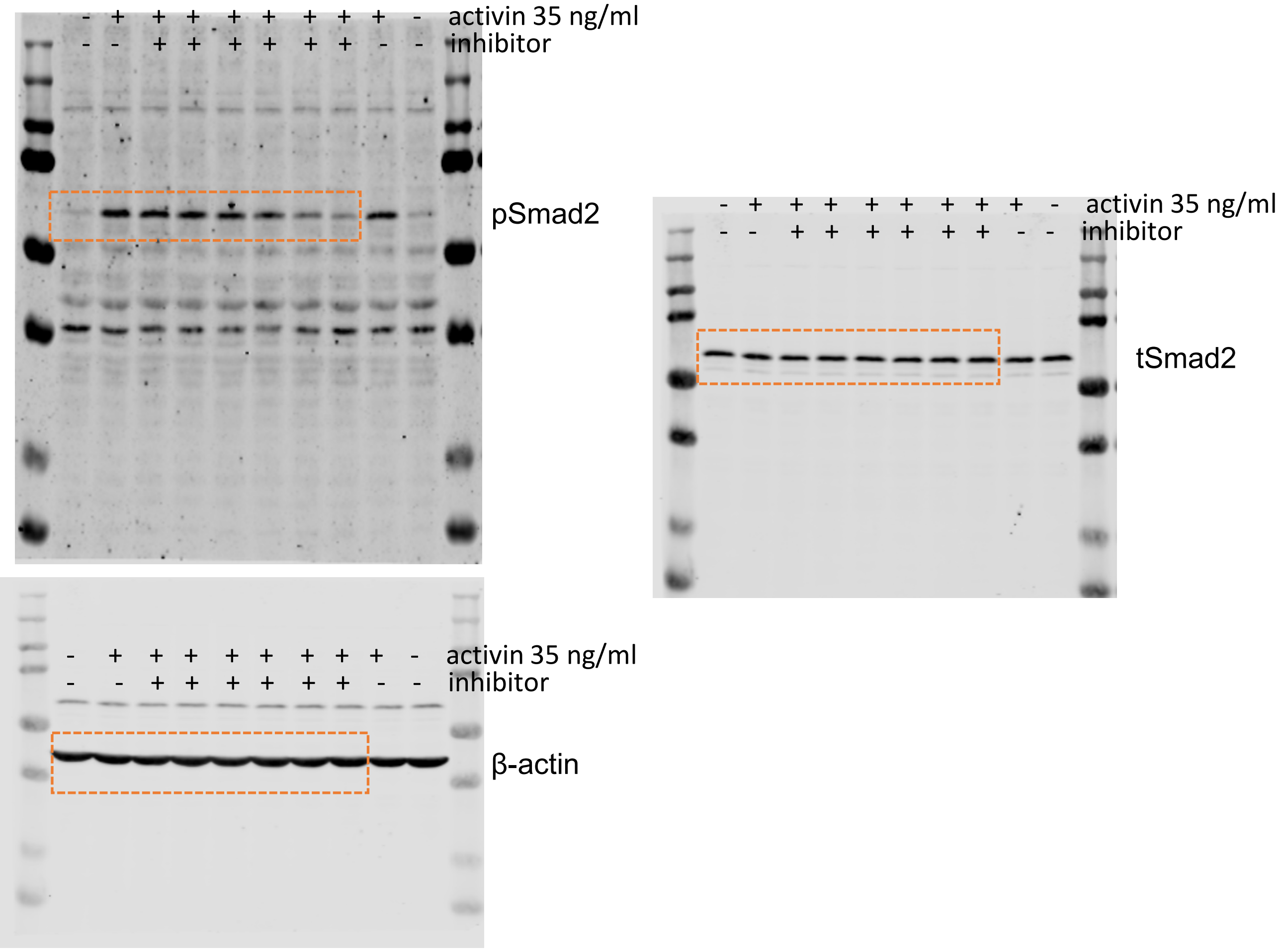

SP Fig 9B

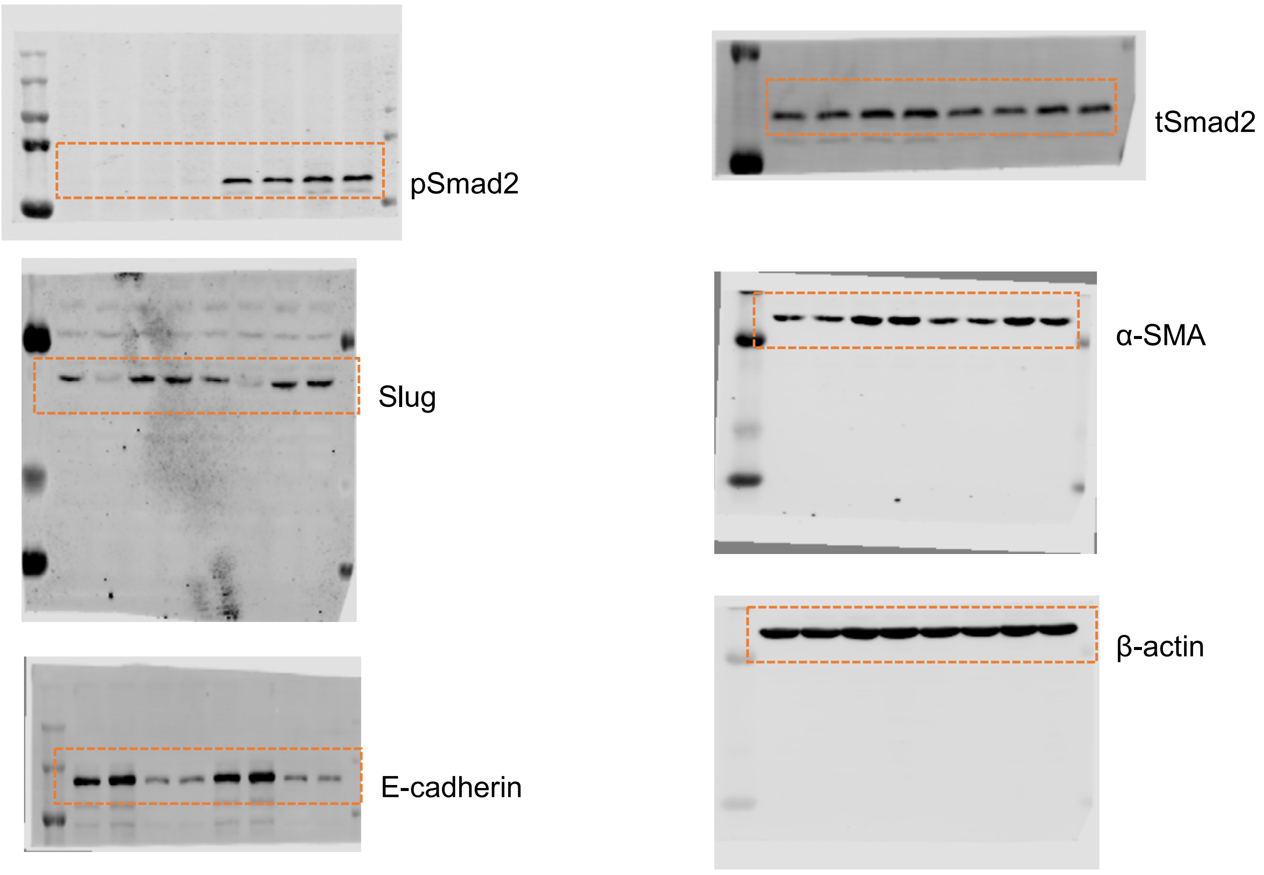

SP Fig 10A

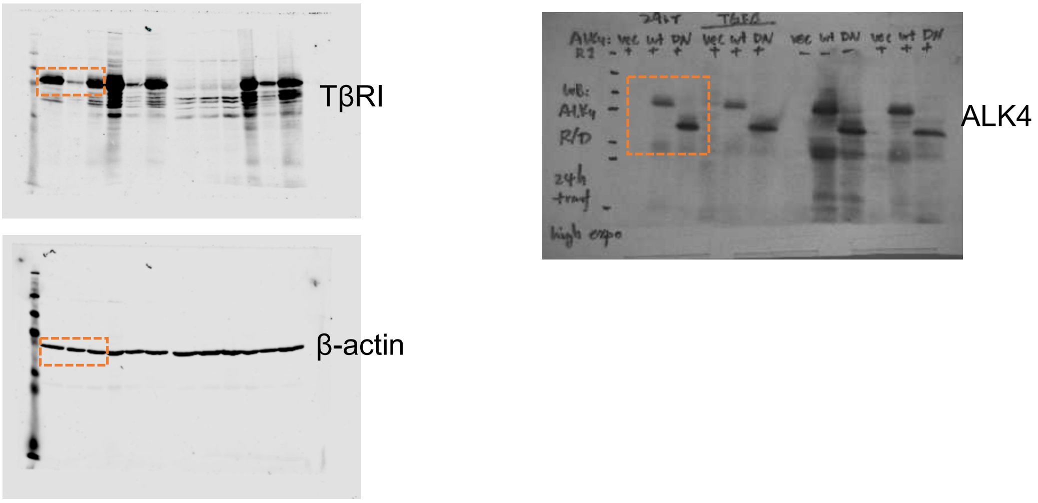

SP Fig 11B

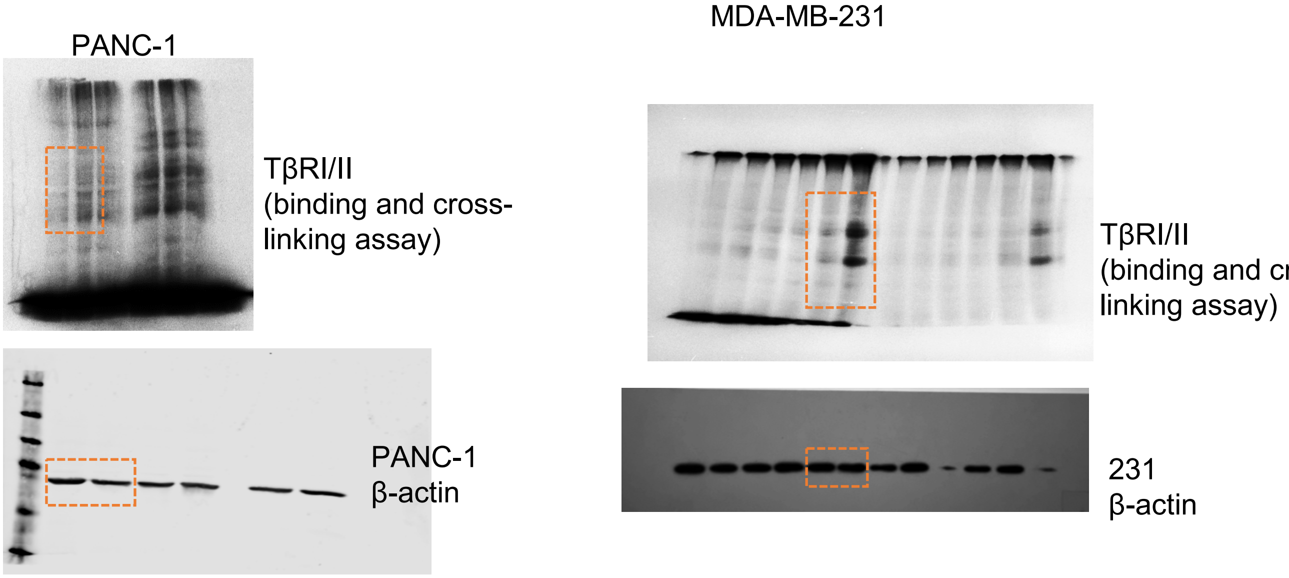

SP Fig 11C

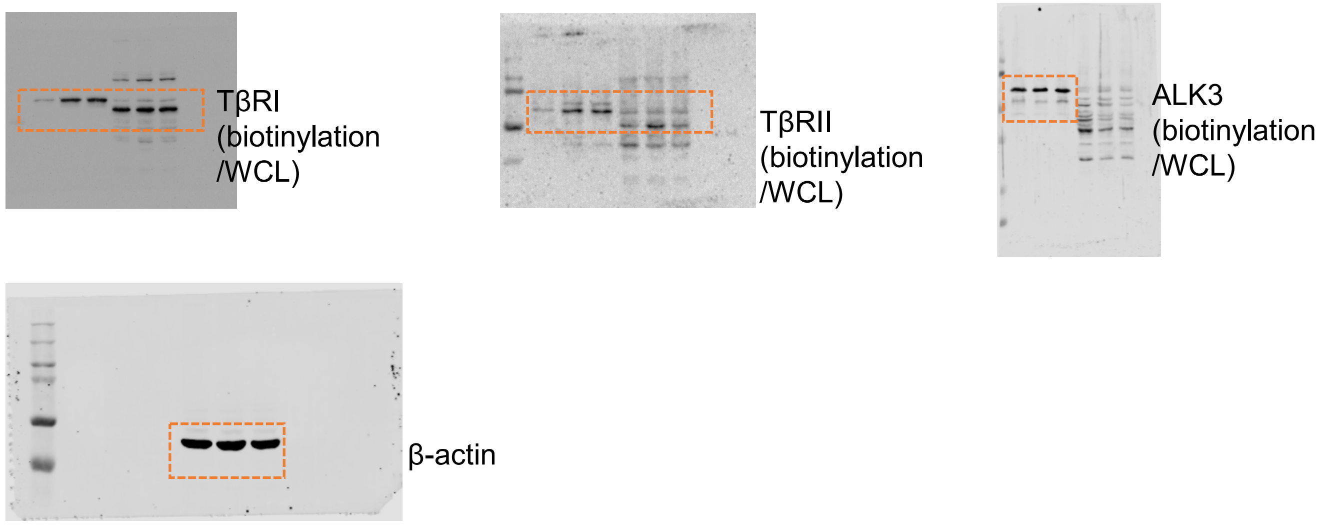

SP Fig 11D

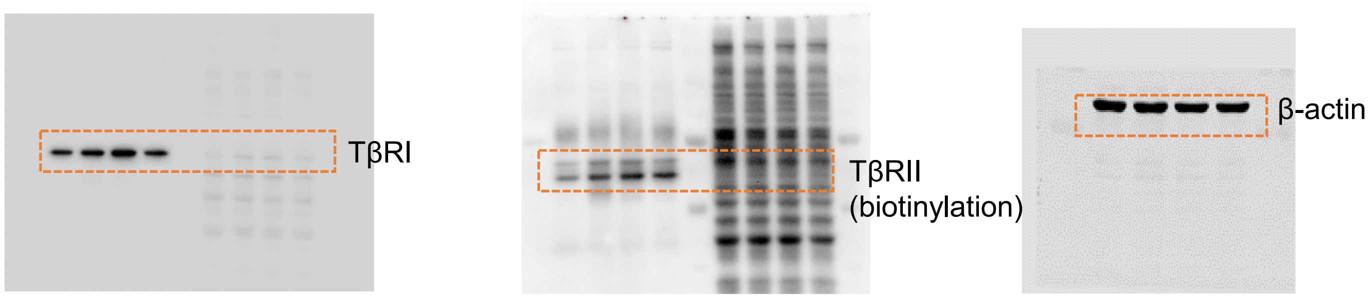

SP Fig 11E

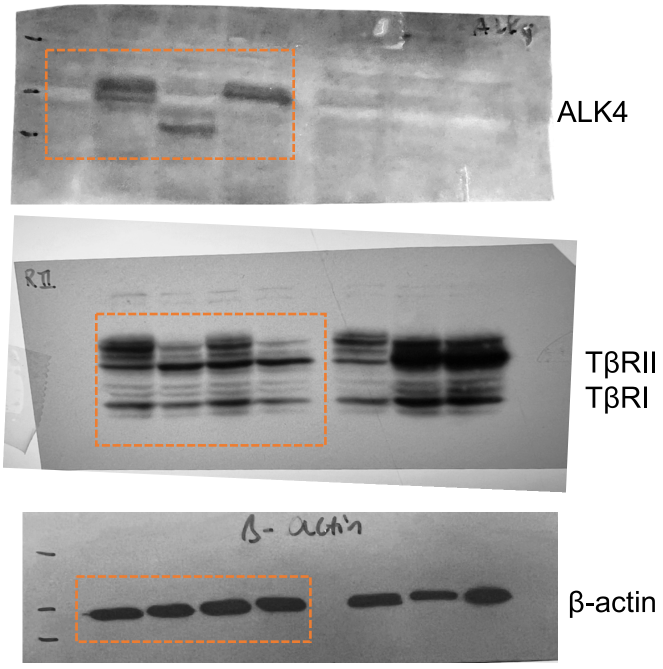

SP Fig 13A

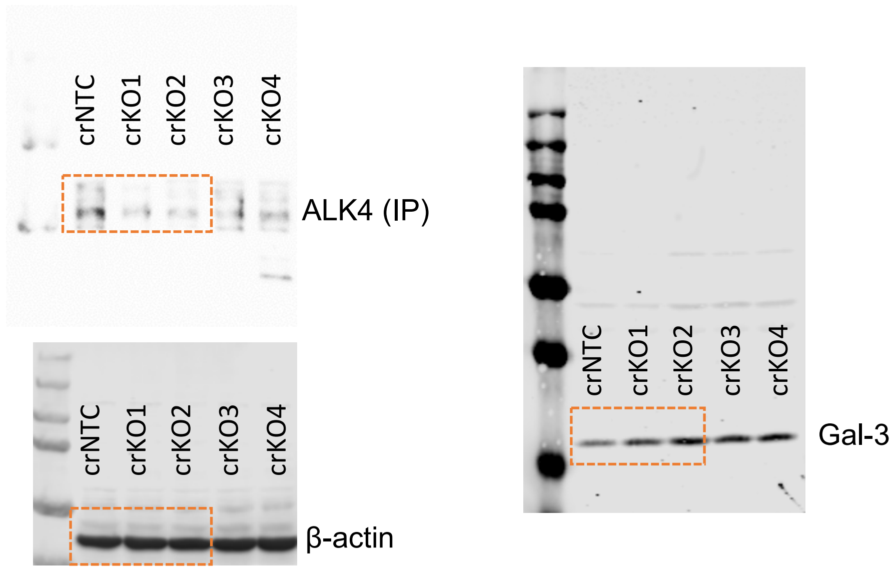

SP Fig 13G

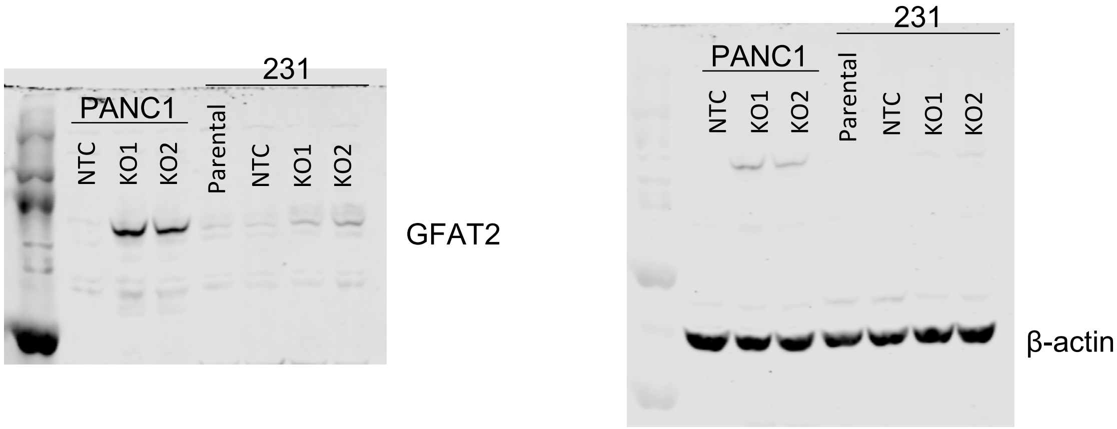

SP Fig 14D

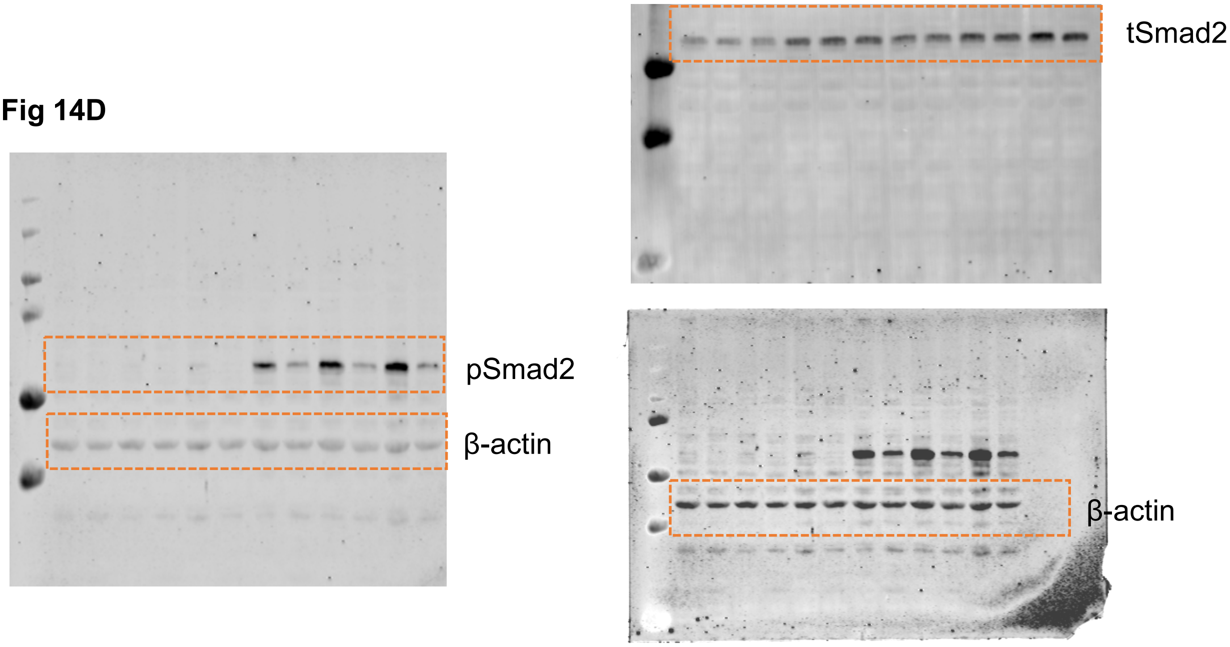

SP Fig 14g

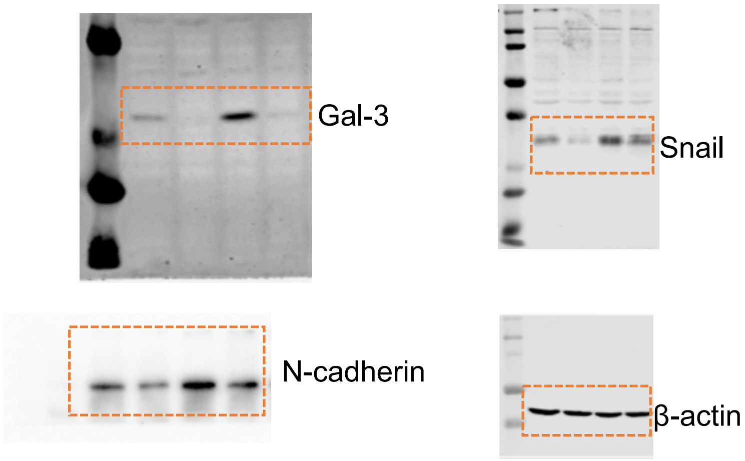

SP Fig 14h

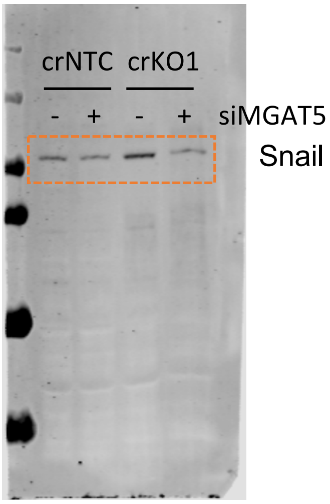

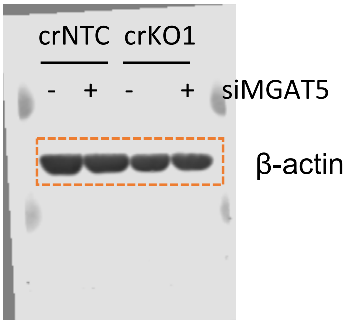

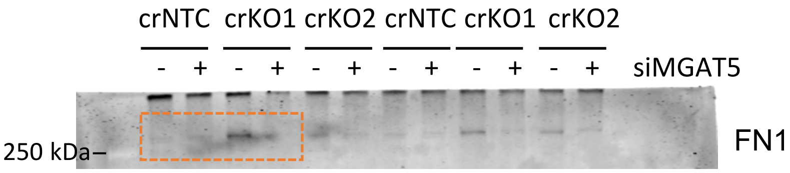

SP Fig 15G

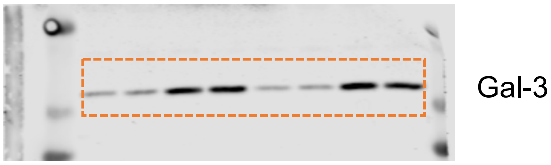

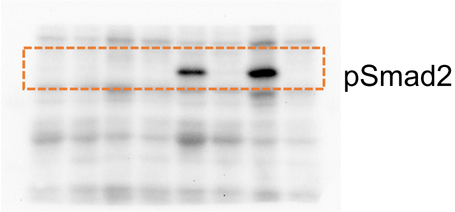

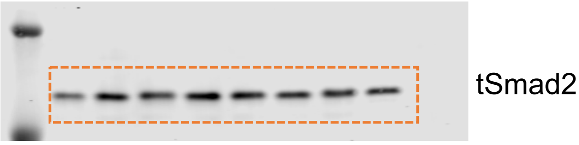

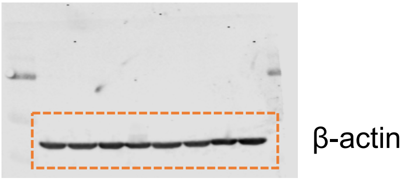

SP Fig 15J

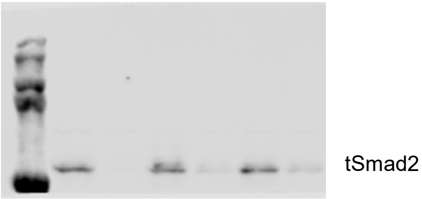

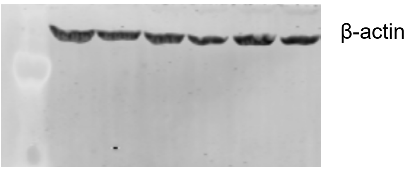

SP Fig 16A

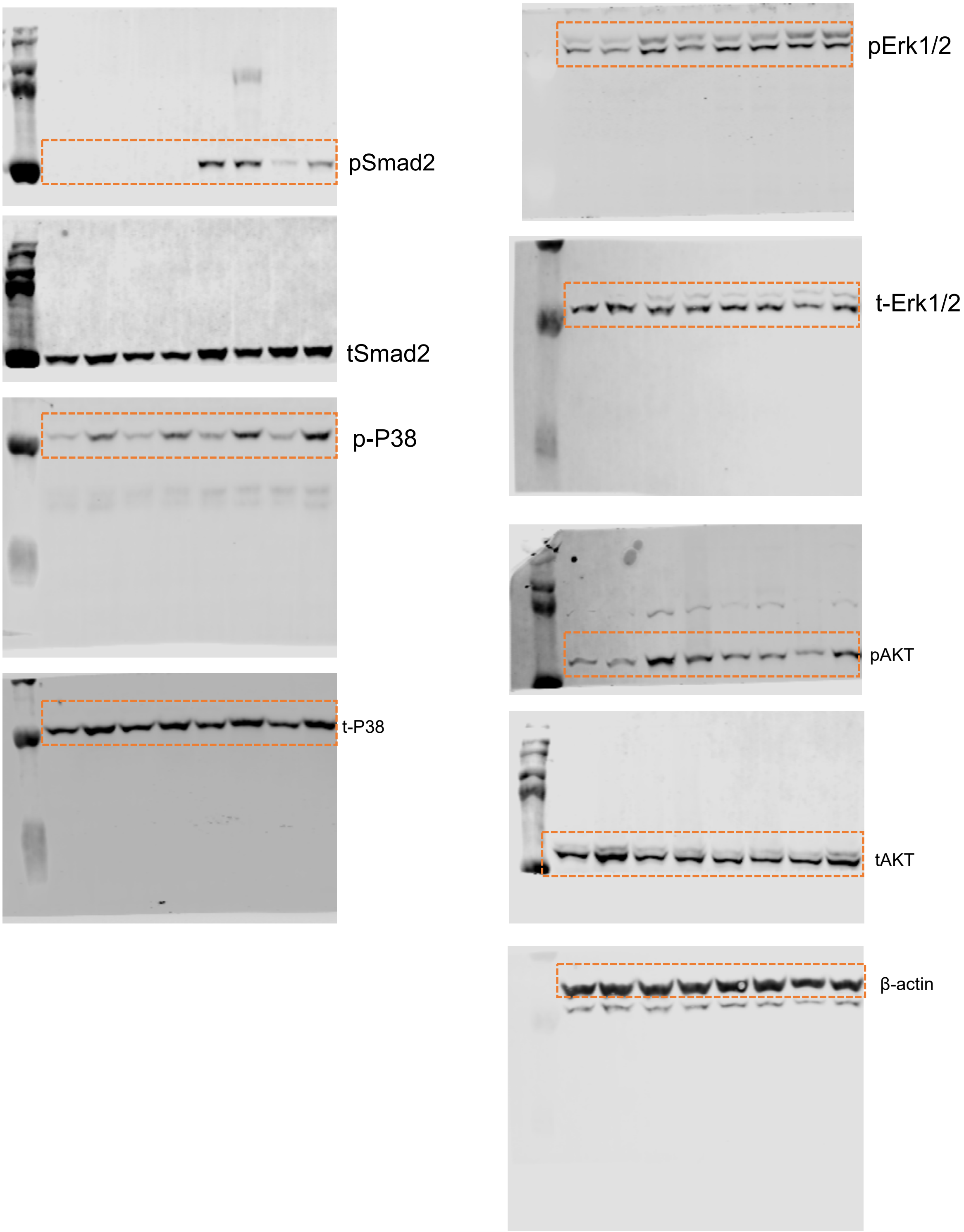

SP Fig 16D

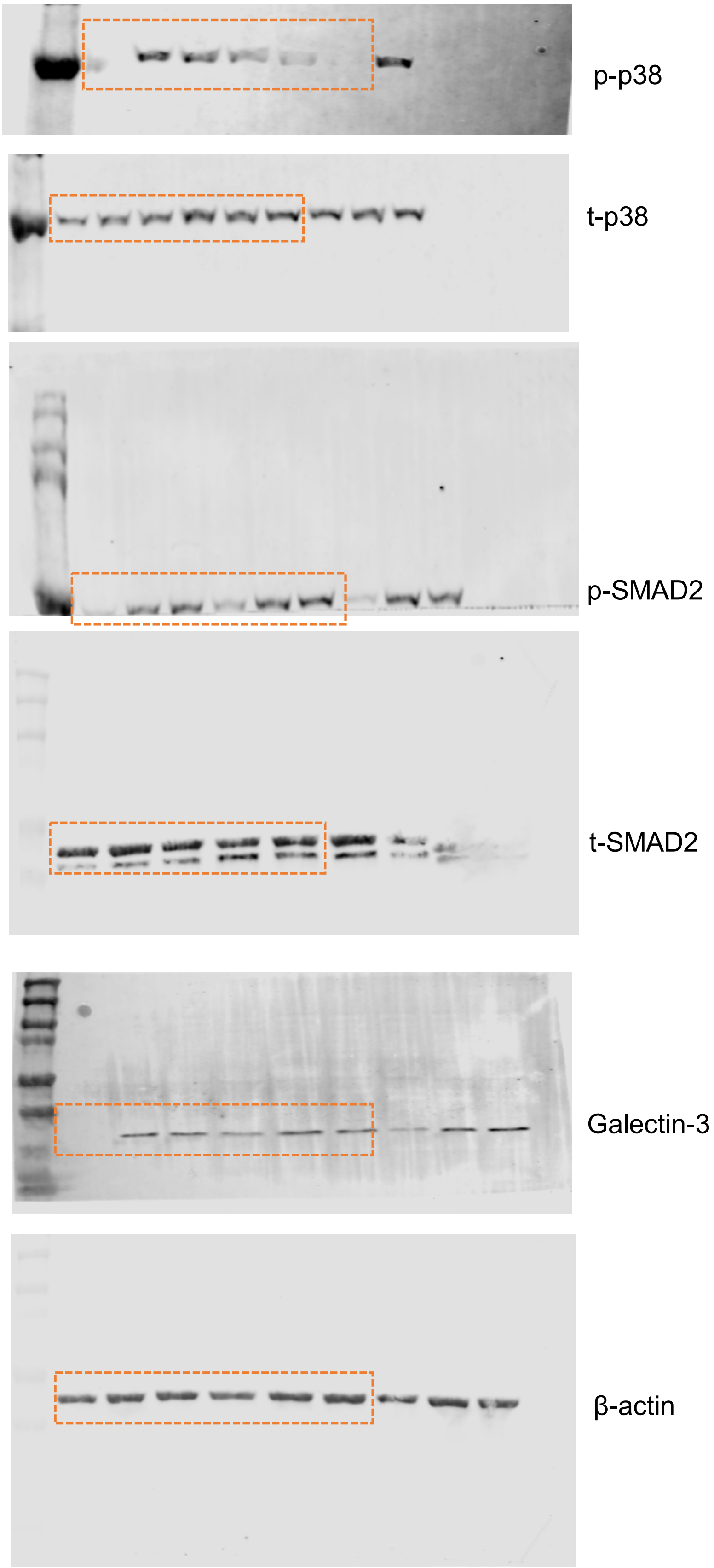

SP Fig 18C

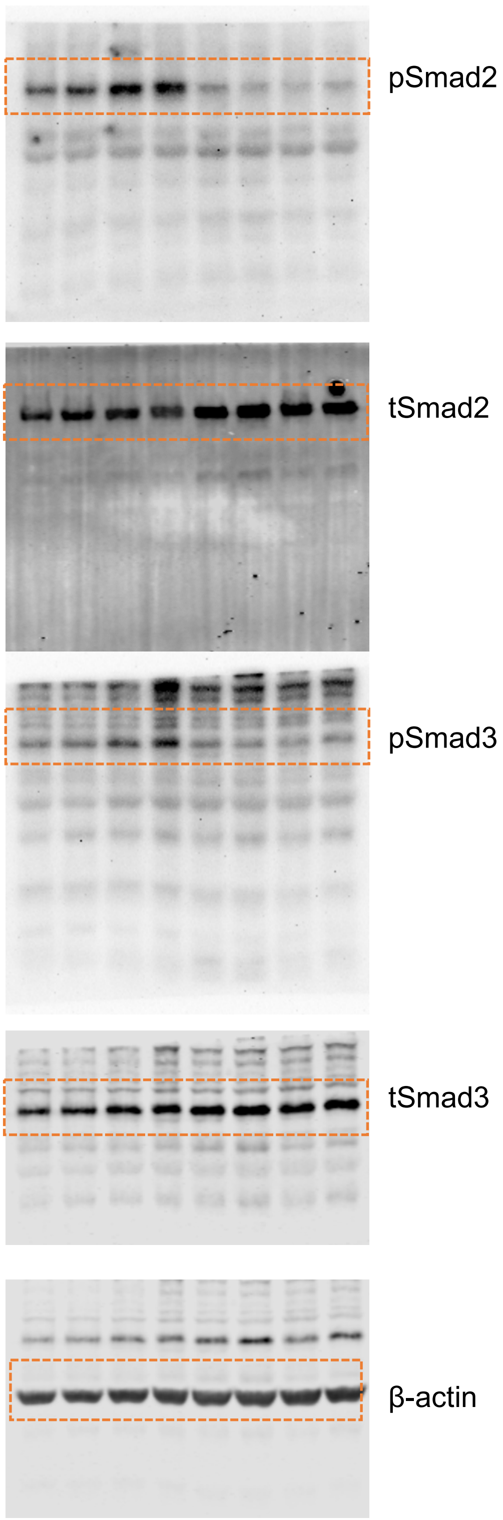

SP Fig 8D

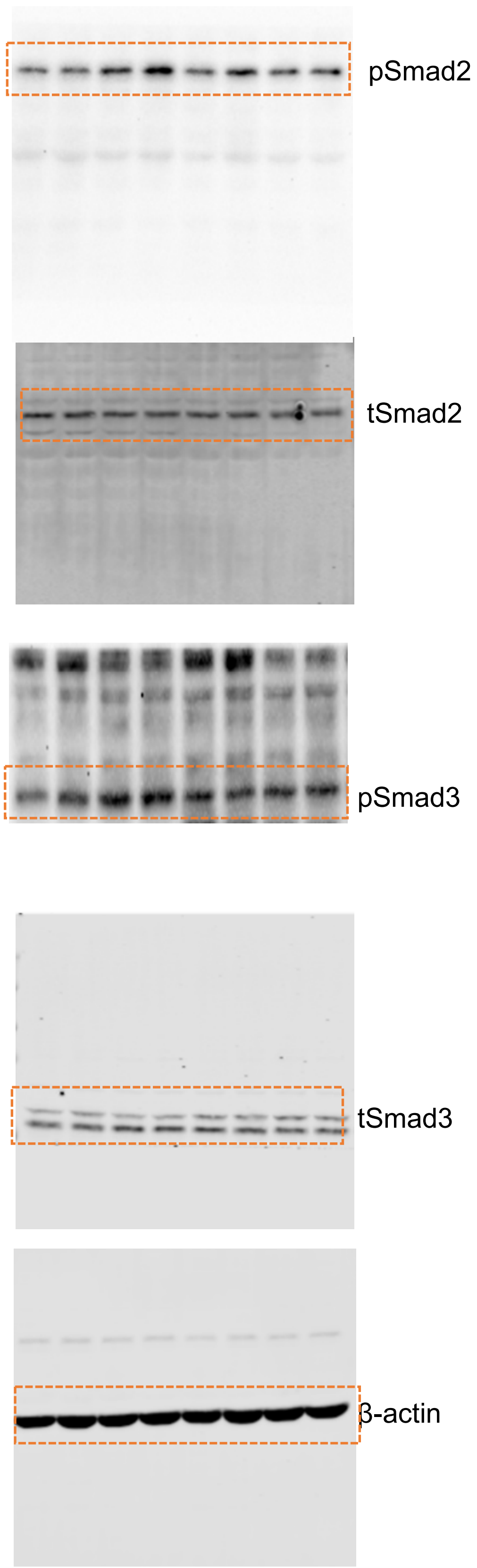

Supplement: Supplementary file 8 — Source data [file 41467_2025_67563_MOESM8_ESM.zip › Source Data/Source data 2.pdf]
